# *Trichinella spiralis* -induced immunomodulation signatures on gut microbiota and metabolic pathways in mice

**DOI:** 10.1371/journal.ppat.1011893

**Published:** 2024-01-02

**Authors:** Xi-Meng Sun, Chun-Yue Hao, An-Qi Wu, Ze-Ni Luo, Saeed El-Ashram, Abdulaziz Alouffi, Yuan Gu, Sha Liu, Jing-Jing Huang, Xin-Ping Zhu

**Affiliations:** 1 Department of Medical Microbiology and Parasitology, School of Basic Medical Sciences, Capital Medical University, Beijing, China; 2 Zoology Department, Faculty of Science, Kafrelsheikh University, Kafr El-Sheikh, Egypt; 3 College of Life Science and Engineering, Foshan University, Foshan, Guangdong province, China; 4 King Abdulaziz City for Science and Technology, Riyadh, Saudi Arabia; QIMR Berghofer Medical Research Institute, AUSTRALIA

## Abstract

The hygiene hypothesis proposes that decreased exposure to infectious agents in developed countries may contribute to the development of allergic and autoimmune diseases. *Trichinella spiralis*, a parasitic roundworm, causes trichinellosis, also known as trichinosis, in humans. *T*. *spiralis* had many hosts, and almost any mammal could become infected. Adult worms lived in the small intestine, while the larvae lived in muscle cells of the same mammal. *T*. *spiralis* was a significant public health threat because it could cause severe illness and even death in humans who eat undercooked or raw meat containing the parasite. The complex interactions between gastrointestinal helminths, gut microbiota, and the host immune system present a challenge for researchers. Two groups of mice were infected with *T*. *spiralis* vs uninfected control, and the experiment was conducted over 60 days. The 16S rRNA gene sequences and untargeted LC/MS-based metabolomics of fecal and serum samples, respectively, from different stages of development of the *Trichinella spiralis*-mouse model, were examined in this study. Gut microbiota alterations and metabolic activity accompanied by parasite-induced immunomodulation were detected. The inflammation parameters of the duodenum (villus/crypt ratio, goblet cell number and size, and histological score) were involved in active inflammation and oxidative metabolite profiles. These profiles included increased biosynthesis of phenylalanine, tyrosine, and tryptophan while decreasing cholesterol metabolism and primary and secondary bile acid biosynthesis. These disrupted metabolisms adapted to infection stress during the enteral and parenteral phases and then return to homeostasis during the encapsulated phase. There was a shift from an abundance of *Bacteroides* in the parenteral phase to an abundance of probiotic *Lactobacillus* and Treg-associated-*Clostridia* in the encapsulated phase. Th2 immune response (IL-4/IL-5/IL-13), lamina propria Treg, and immune hyporesponsiveness metabolic pathways (decreased tropane, piperidine and pyridine alkaloid biosynthesis and biosynthesis of alkaloids derived from ornithine, lysine, and nicotinic acid) were all altered. These findings enhanced our understanding of gut microbiota and metabolic profiles of *Trichinella* -infected mice, which could be a driving force in parasite-shaping immune system maintenance.

## Author summary

The complex interactions between gastrointestinal helminths, gut microbiota, and the host immune system are unknown. Here, we studied stool and serum samples from *Trichinella*-infected mice at different stages of development by 16S rRNA gene sequencing and non-targeted LC/MS metabolomics. We found that there is abundant *Bacteroides* in the gut microbiota during the parenteral phase of *Trichinella* infection, and the encapsulated phase shifts to a large number of probiotics *Lactobacillus* and Treg-associated-*Clostridia*. Metabolic activities also change in the enteral and parenteral phases, such as abnormal cholesterol metabolism. It is accompanied by changes in the Th2 immune response, lamina propria Treg, and immune hypo-responsiveness metabolic pathways. These findings contribute to a better understanding of the complex interactions between gastrointestinal helminths, gut microbiota, and host immune system. And the system-level knowledge of parasite metabolism and gene regulation through multi-omics joint analysis can help identify new targets for anthelmintic drugs and develop metabolite-based diagnostic tools.

## Introduction

The hygiene hypothesis assumes that the decreased incidence of infections with parasites and bacteria in developed countries may cause immune system deregulation and the consequent development of allergic and autoimmune diseases [1]. By neutralizing immunological pathways that would normally expel them and reset the limits of immunological response, helminths may effectively establish themselves inside the mammalian body. They also reduced responses to unrelated allergens and autoantigens, which may benefit the host [2,3]. Helminth-induced immunomodulation signatures on autoimmunity suppression fall into two categories: those that limit Th1 and Th17 responses through regulatory T cell (Treg) induction and those that activate helminth-specific type 2 responses (Th2). There is now a substantial body of experimental evidence supporting the role of gastrointestinal (GI) helminth parasites as immune regulators because of their ability to skew host immune responses toward Th2/Treg phenotypes [4–11]. Infection with helminths induces polarized type 2 immunity characterized by elevated expression of cytokines, including IL-4, IL-5, IL-10, and IL-13 [12,13].

Accumulating evidence supports the hypothesis that gastrointestinal (GI) helminth infections are associated with quantitative and qualitative modifications in the gut commensal microbiota with different downstream effects on parasite-mediated immune modulation, which can influence immune regulation and control of harmful inflammation [14–20]. The immune-molecular mechanisms underlying host-parasite–microbiota interactions remain elusive. Infections with GI helminths have been shown to affect the composition of the host gut microbiota and the relative abundance of individual microbial species, with likely downstream effects on host immunity and metabolic potential. Infections with gastrointestinal helminths have been shown to alter the gut microbiota composition and the relative abundance of individual microbial species [16]. These changes may impact host immunity and metabolism [17]. Fecal microbiota transplants from *Heligmosomoides polygyrus*-infected to naïve mice were enough to induce activation of regulatory T cells (Tregs) in the naïve mice and modulate host responses to concomitant diseases, ultimately affecting their clinical outcome [19,21]. *Trichuris muris* infections directly affected the composition of the caecal gut flora, comprising expanded populations of *Firmicutes*, *Actinobacteria*, *Deferribacteres*, and *Proteobacteria*, and a concomitant reduction in *Bacteroidales* [22]. In addition, selected populations of gut microbes may indirectly assist the establishment, or the elimination, of selected helminth parasites via immune-mediated events. Administration of *Lactobacillus* species to mice before experimental infection with *H*. *polygyrus* resulted in substantially increased worm burdens, which were mediated by bacterial-induced expansions of populations of Treg cells [23]. Recent studies had shown that parasite-elicited Th2 immune responses played a key role in helminth-associated qualitative and quantitative gut microbiota changes since such changes were not observed in Th2-deficient knock-out mice [24,25]. The host type 2 responses to parasite infection led to a depletion of the intestinal segmented filamentous bacteria (SFB), which reduced the expression of the pro-inflammatory cytokine IL-17 in the intestine [25].

While still in its infancy, the application of metabolomics to the study of helminth infections is rapidly emerging as a fertile approach to better understand host-parasite interaction, especially on the aspect of helminth-induced immunomodulation. Changes in the metabolomic profiles of host fluids and tissues in response to parasite infection are critical for understanding parasite pathogenesis and parasite-induced immune responses and show great promise for diagnostics [26]. Metabolomic analysis of host samples (plasma, urine, or stool) has been widely used to identify biomarkers of parasitic helminth infection [27–29], which can distinguish infected from uninfected animals, often early in the disease’s course, by using either abundance changes in the metabolomic profile or unique metabolites to infected animals. There is a shared finding among these studies of significant changes in metabolites produced by the intestinal flora following infection with intestinal and extra-intestinal helminths. This shows that changes in the intestinal flora’s composition can significantly affect the host’s metabolite profile [29,30]. It has been reported that protozoan infections can alter host metabolism. These studies provided novel insights into disease pathogenesis and infection responses [31,32]. The urine and plasma samples of *Plasmodium berghei*-infected mice showed an upregulation of glycolysis, increased energy demand, and disruption of the gut microbiota [33–35]. Many of the intermediates involved in amino acid and energy metabolism were altered in mouse sera and brain samples after infection with *Toxoplasma gondii* [36,37].

As a multicellular parasitic helminth, *T*. *spiralis* is an interesting infection model for studying the induction of type 2 responses and Treg cells in response to infection. During *T*. *spiralis* infection, the entire life cycle is completed in the same host, sequentially containing adult, newborn larval (NBL), and muscle larval (ML) stages. The release of NBLs from sexually mature adult worms in the intestine is followed by their migration and the eventual establishment of a chronic infection in which MLs are present in the host’s skeletal muscles [38]. Each of these life stages may have a distinct impact on the host’s immune response. These characteristics make the *T*. *spiralis*-mouse system a good model for studying host-parasite relationships. This study used the *T*. *spiralis*-mouse model to investigate how gut microbiota and metabolic pathways support immunomodulation signatures induced by helminths. An integrated experimental design investigated *T*. *spiralis* infection-induced type 2 immunity and lamina propria resident Treg cells. Using 16S rRNA gene sequence and gas chromatography/mass spectrometry (LC/MS)-based metabolomic analyses, we investigated the effect of *T*. *spiralis* infection on intestinal microbiota and serum metabolism of infected animals. A time-course experiment was undertaken to monitor mice’s changes in intestinal microbiota and the effects on metabolomic profiles. This is one of the first studies to integrate helminth immunomodulation, gut microbiome, and serum metabolic profiles in mice with a bioinformatic approach to identify inter-relationships between host, gut microbiota, and metabolism in mice.

## Results

### *T*. *spiralis* induces duodenal lesions and inflammation

**Fig 1:**
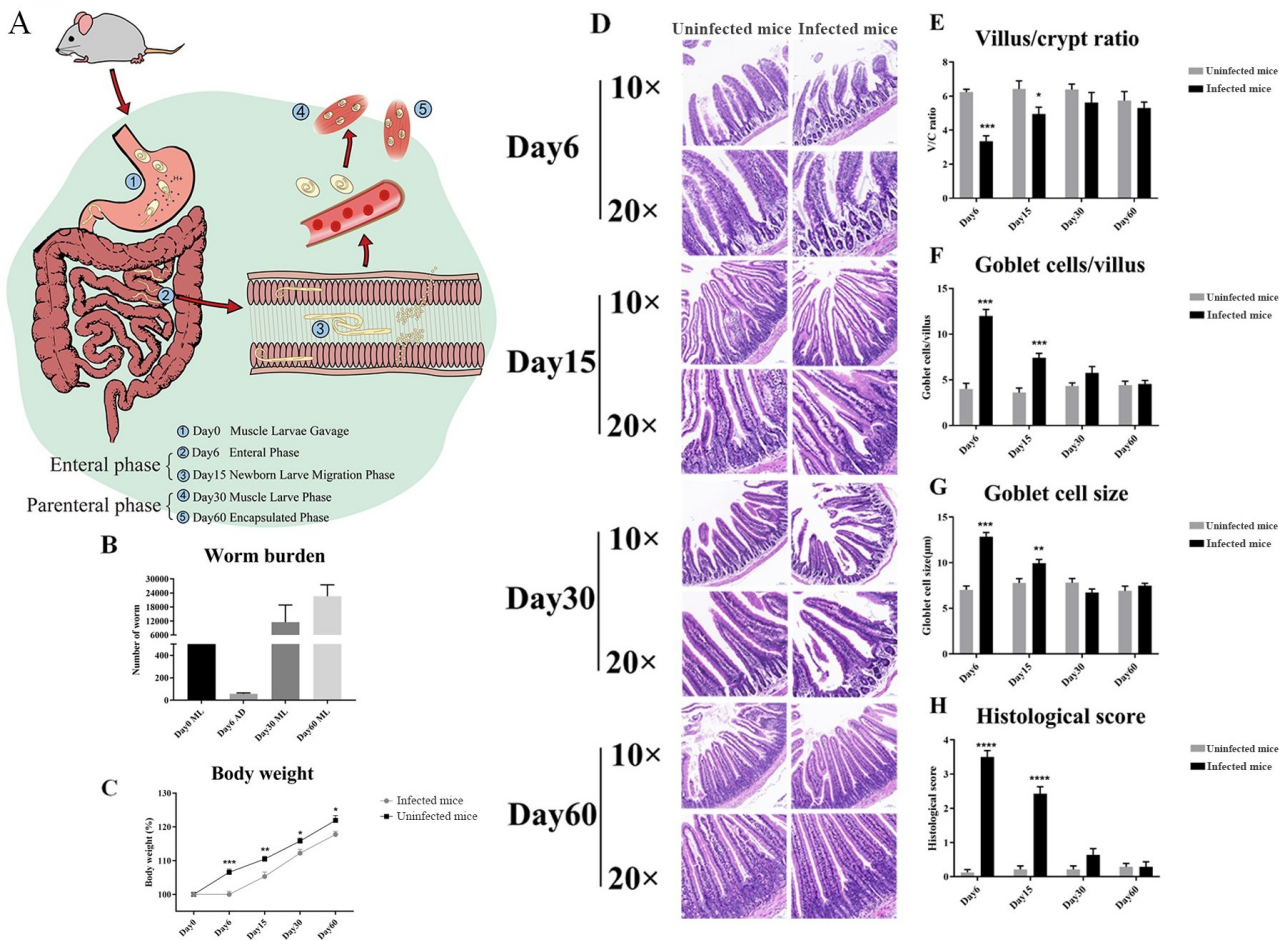
Sampling Strategy and Inflammation Index of *T*. *spiralis* Infection. (A) Infection and sampling strategy. (B) Physiological Changes. The number of worms observed in the small intestines and muscles of individual *T*. *spiralis* infection mice at each sampling time point (n = 5). (C) Body weights of infected (n = 7) and uninfected mice at each sampling time (n = 7). (D) H&E-stained duodenum represents sections from infected and uninfected mice at each time point (original magnification: ×10 or ×20). (E) Villus height to crypt depth (villus/crypt ratio). (F) The number of goblet cells per villus. (G) Goblet cell size. (H) Histological score (n = 6). *p < 0.05, **p < 0.01, ***p < 0.001.

Wild type mice were randomly divided into infected and uninfected (control) groups. We administered 400 muscle larvae (ML) to mice via gavage (day 0) to quantify the effects of *T*. *spiralis* infection in mice. We then monitored the infection’s progressive effects on worm burden, weight loss, and duodenal pathology on day 6, 15, 30, and 60 after gavage. Based on the life cycle (Fig 1A), the larvae invaded the small bowel mucosa on day 6 after gavage, which developed into adult worms (AD). After incubation, approximately 70 adult worms entered the small intestine on day 6 of the enteral phase (day 6). In the parenteral phase (day 15), females released newborn larvae that reached the bloodstream and lymph drainage system. Approximately 15,000 larvae migrated to the striated muscles where they encyst (day 30), and the larval burden reached its peak in the encapsulated phase (day 60). (Fig 1B). These results were consistent with the life cycle of *T*. *spiralis* in mice. Slowly rising of body weight was a common symptom of a severe *T*. *spiralis* infection. Infected mice increased slowly in body weight compared to uninfected controls following worm burden (Fig 1C). These differences were consistent in all life stages, showing that *T*. *spiralis* infection impacts body weight increasing.

We collected duodenal tissue on days 6, 15, 30, and 60 after gavage. The pathological section results suggested that the duodenum of infected mice had mucosal lesions compared with uninfected mice (Fig 1D). Histological morphometry suggested a decreased ratio of villus height to crypt depth (Fig 1E), an increased number of goblet cells per villus (Fig 1F), goblet cell size (Fig 1G), and histological score (Fig 1H) in infected mice compared with uninfected mice. The duodenum was scored (0–3) for both inflammation and edema. These gut damage parameters of the duodenum indicated that the most severe pathological changes were observed in the enteral phase and were most often confined to the lamina propria and epithelium, resolved by day 30.

### *T*. *spiralis* infection induces type 2 immunity and enhances the frequency of Tregs in mesenteric lymph node

**Fig 2:**
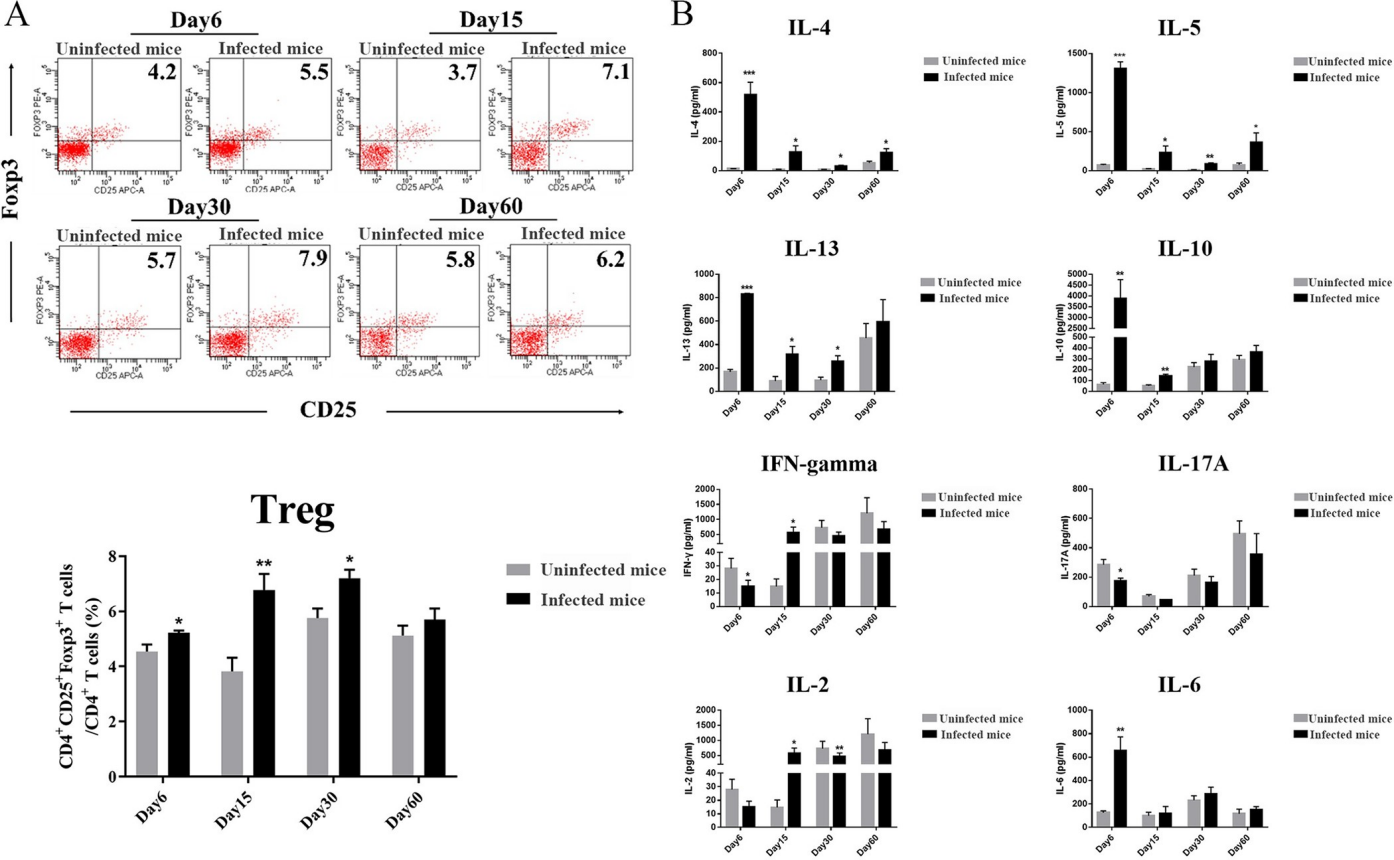
Cytokine Profiles and the Differentiation of Treg Cells Measured by Mesenteric Lymph Nodes Isolated from *T*. *spiralis* Infected Mice. On Days 6, 15, 30, and 60 after infection with *T*. *spiralis*, five infected mice at each time point were sacrificed, and MLNs were isolated. (A) The cells were sorted with CD3, CD4, CD25, and Foxp3 by FACS, and the percentage of CD25^+^ Foxp3^+^ cells in the CD4^+^ T cells population was shown. (B) The culture supernatants were collected after being incubated with α-CD3/CD28 for 48 h, and the concentrations of IL-4, IL-5, IL-10, IL-13, IL-2, IFN-γ, IL-17A, and IL-6 were measured by ELISA. Data are from three independent experiments expressed as mean SEM, with * P < 0.05, ** P < 0.01, and *** P< 0.001 compared to the uninfected group.

This study aimed to examine the *T*. *spiralis* infection-induced immune response by measuring the Th2 cell cytokine concentrations of IL-4, IL-5, IL-13, and IL-10 in response to restimulation with anti-CD3 and anti-CD28 mAbs, and the frequency of Treg cells by mesenteric lymph node (MLN) at each time point. The study found elevated IL-4, IL-5, IL-13, and IL-10 in infected mice on day 6, which remained until day 30 and returned to normal levels by day 60 (Fig 2B). The percentage of Treg cells in the mesenteric lymph nodes was also higher in infected mice, peaking on day 15 and returning to normal levels by day 60 (Fig 2A). In contrast, after restimulation with anti-CD3 and anti-CD28 mAbs, infection-induced mouse MLN cells secreted low levels of Th1 cell cytokines IL-2 and IFN-γ. This response only increased on day 15. We also found that the level of IL-17A decreased while IL-6 increased on day 6. *T*. *spiralis* infection results in a Type-2 immune response.

### Expansion of Tregs in lamina propria of the colon during *T*. *spiralis* infection

**Fig 3:**
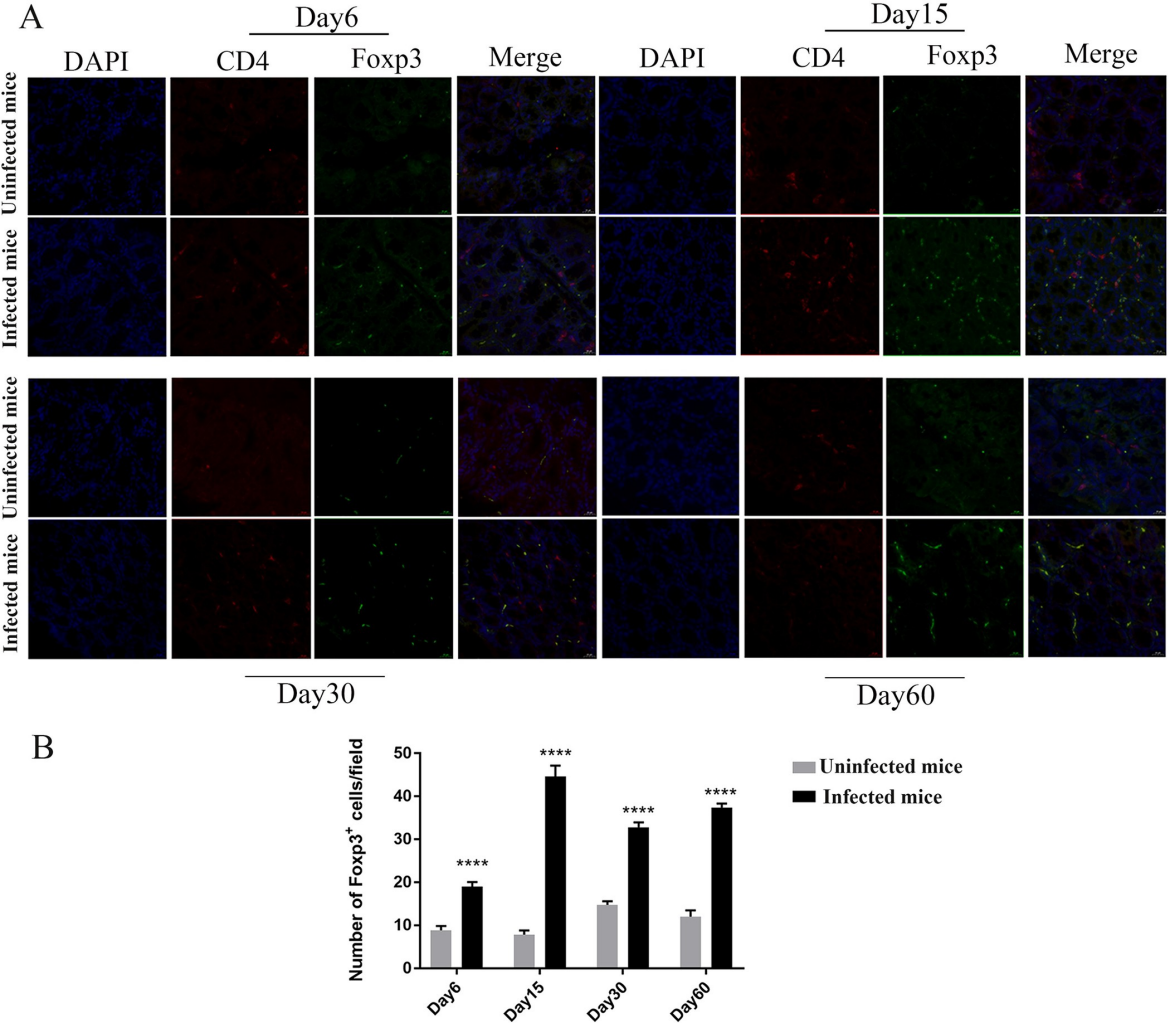
*T*. *spiralis* Infection Enhanced Residential Tregs in Lamina Propria of the Large Intestine. Forty-eight Foxp3^EGFP^ mice were randomly assigned to infected (N = 24) and uninfected (N = 24) groups. On Days 6, 15, 30, and 60 after infection with *T*. *spiralis*, 6 infected or uninfected mice at each time point were sacrificed, and the colon was isolated. (A) Immunofluorescence analysis of Foxp3 expression. It was mainly observed on CD4^+^ Treg (×400 magnification). EGFP, Enhanced Green Fluorescent Protein, Cy3, indocarbocyanin red, and DAPI, 4′,6-Diamidino-2- phenylindoldihydrochlorid blue–nuclear counterstaining. (B) The number of Foxp3^+^ cells per field.

To monitor the Treg cells during *T*. *spiralis* infection, 48 Foxp3^EGFP^ mice were randomly assigned to infected (N = 24) and uninfected (N = 24) groups. Twenty-four Foxp3^EGFP^ mice were infected with 400 muscle larvae of *T*. *spiralis* via the gastrointestinal tract. At each time point, 6 infected or uninfected mice were sacrificed and the lamina propria of the colon were isolated. Foxp3^+^ regulatory T cells in the lamina propria of the colon were identified and analyzed by EGFP/CD4 immunohistochemistry. We stained CD4 with CD4-Cy3 antibody (red light) and nuclei with DAPI (blue light). Foxp3 in mice had its own EGFP green fluorescence, and an automatic slide scanner was used for fluorescence mode scanning. We found that infected colon lamina propria showed a higher accumulation of CD4^+^ Foxp3^+^ Treg cells, which were sustained by the encapsulated phase (Fig 3).

### Changes in the gut microbiome in *T*. *spiralis-* infected mice

**Fig 4:**
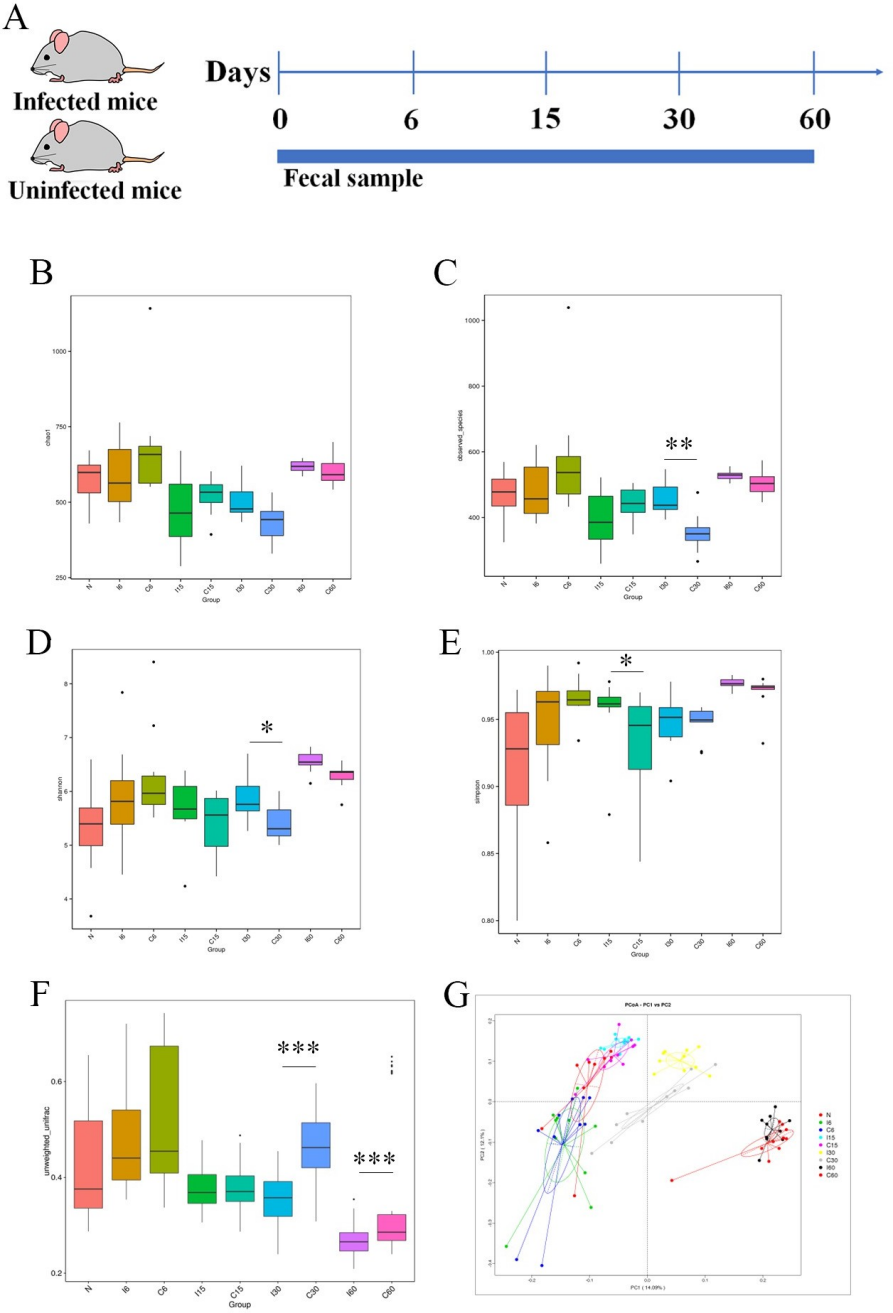
Infection with *T*. *spiralis* modulates the colon microbiota in mice. The experimental design of murine infection, as well as the taxonomic and α and β diversity of gut microbiomes on days 0, 6, 15, 30, and 60 after infection with *T*. *spiralis*, ten infected mice at each time point were sacrificed, and the feces were collected. The α diversity refers to the diversity within a sample, while β diversity refers to the diversity between samples. (A) The murine infection experimental design. (B) Comparison of Chao 1 index (an α diversity indicator used to estimate the total number of microbial species) between infection and control groups. (C) Comparison of observed species between two groups. (D) Comparison of the Shannon index (a measure of diversity considering the number and abundance of species in a sample) between two groups. (E) Comparison of Simpson index (a measure of diversity considering the number of species and their relative abundance) between two groups. (F) Unweighted pairwise UniFrac distances were averaged within each group to calculate an average diversity value (a proxy for inter-individual variation). (G) PCoA plot based on the unweighted UniFrac distance (to compare whether there were significant differences in microbial communities in specific evolutionary lineages of samples) of gut microbiota by positioning each sample from infected vs. uninfected group (I6 vs. C6 group, *P* = 0.3631; I15 vs. C15 group, *P* = 0.7912; I30 vs C30 group, *P* = 0; I60 vs. C60 group, *P* = 0). N, represented the result of the samples collected before *T*. *spiralis* infection on day 0. Median, dispersion degree, maximum, minimum, outliers and were shown as boxes. *P <0.05, **P <0.01, ***P <0.001. *P* values are from Wilcox test.

The study investigated the alterations in the gut microbiome of infected mice by sequencing the fecal 16S rRNA V4 region. The overall experimental design of the study was summarized in Fig 4A, which provided an overview of the methods used and the study’s timeline. We used various diversity measures to compare infected mice’s gut microbiota with healthy control mice at different time points (day 0, 6, 15, 30, and 60). The α diversity of the gut microbiota, which referred to the diversity within a sample, was not significantly different between the infected and uninfected groups on day 6 and day 60 (Fig 4A–4G). The Chao1 index, an α diversity indicator used to estimate the total number of microbial species, showed no significant difference at each time point (day 0, 6, 15, 30, and 60) (Fig 4B). However, on day 15, the infected group exhibited a higher Simpson index (Fig 4E), a measure of diversity considering the number of species and their relative abundance. On day 30, the infected group had higher values of observed species (Fig 4C) and a higher Shannon index (Fig 4D), which are also diversity measures considering the number and abundance of species in a sample. But the subtle difference from the Shannon index was that the Simpson index focused more on the relative abundance between different species, while the Shannon index focused more on species richness. To investigate the overall structure of the gut microbiota, we constructed a Principal Coordinate Analysis (PCoA) score plot based on weighted UniFrac distances. Weighted UniFrac distances was used to compare whether there were significant differences in microbial communities in specific evolutionary lineages of samples. Thus, PCoA measures the similarity between bacterial communities. The results showed that on days 30 and 60, the structure and composition of the microbiota differed significantly between the infected group and the uninfected group, indicating that the infection had a lasting effect on the gut microbiome. However, colonization with *T*. *spiralis* on days 6 and 15 did not significantly affect β diversity (Fig 4F–4G), which referred to the diversity between samples.

**Fig 5:**
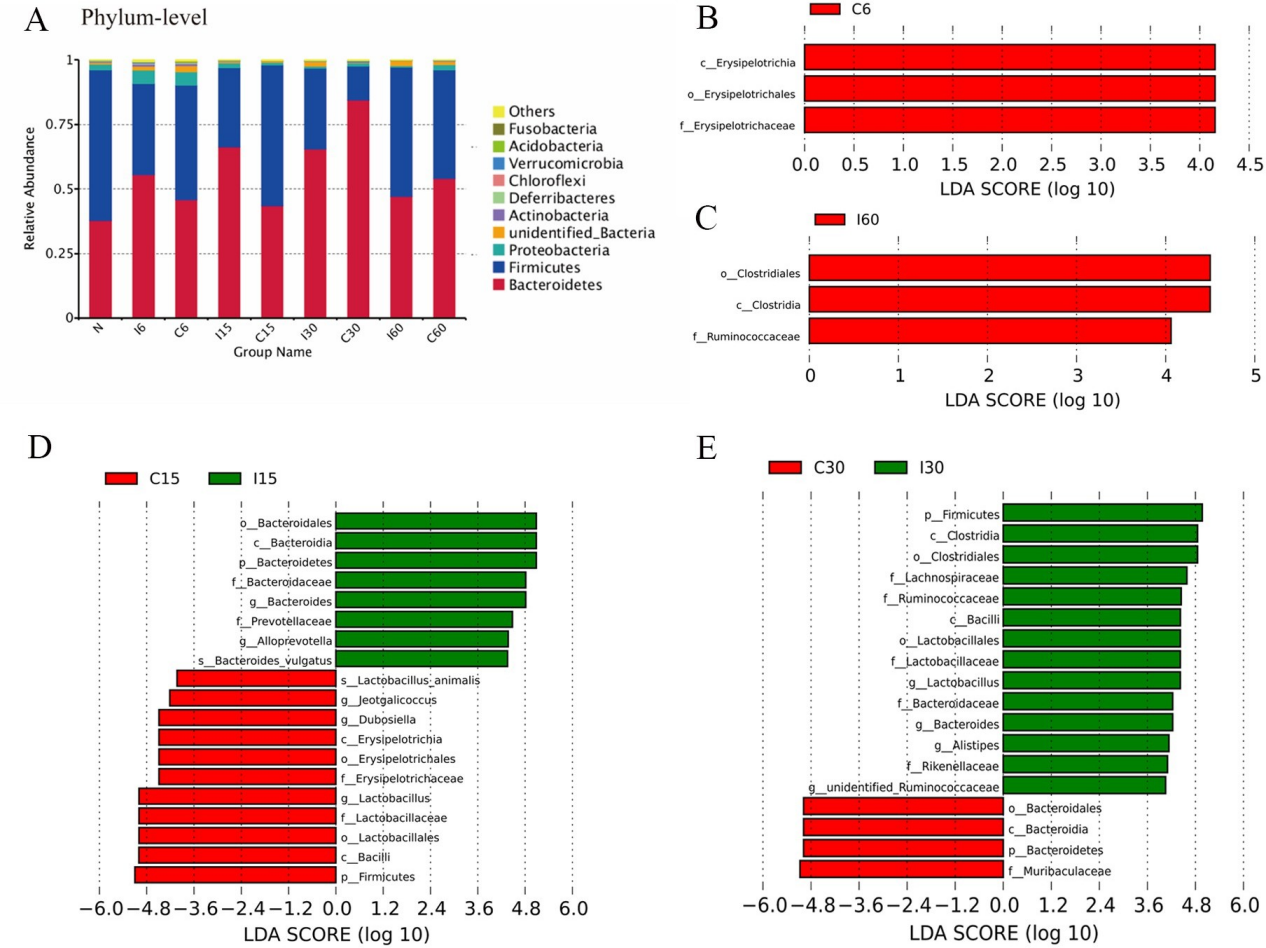
The microbiome composition differences in the infected and uninfected group as well as the log linear discriminant analysis (LDA) effect size quantifies the degree to which each lineage contributes to the uniqueness of two groups. The data showed the abundance of different bacterial groups in the infection and control groups at each time point. For the quantitative microbiome analyses, LEfSe method was used to identify significant bacterial groups between the infected and uninfected groups, which was performed by LEfSe software with a default LDA Score threshold of 4. (A) Community bar plot analysis was shown at the phylum level. N, represented the result of the samples collected before *T*. *spiralis* infection on day 0. (B) C6 group. (C) I60 group. (D) I15 vs. C15 group. (E) I30 vs. C30 group.

Community bar plot analysis (Fig 5) showed that the gut microbiota community composition of the infected group differed significantly from the uninfected group (*P* = 0.235 on day 6; *P* = 0.001 on days 15 and 30; *P* = 0.014 on day 60, *P* value from Adonis analysis). The identified gut microbiota comprised 10 phyla. The most abundant bacterial group was the *Bacteroidetes*, followed by the *Firmicutes*, *Proteobacteria*, *Actinobacteria*, *Deferribacteres*, *Chloroflexi*, *Verrucomicrobia*, *Acidobacteria*, and *Fusobacteria*. The most notable differences were the relatively greater amounts of *Bacteroidales* and the fewer amounts of *Firmicutes* on day 6 and day 15, and they reversed these differences on day 30 and day 60. In particular, *Bacteroidetes* accounted for over 66% of the sequences in the infection group but only about 43% of the sequences in the control group on day 15. In contrast, *T*. *spiralis* colonization was associated with a 2.4-fold increase in the relative number of *Firmicutes* sequences on day 30 (Fig 5A). Additionally, *T*. *spiralis* colonization had similar effects on the *Proteobacteria*, shifting from a 1.7-fold increase on day 15 to a 2.7-fold decrease on day 60.

**Fig 6:**
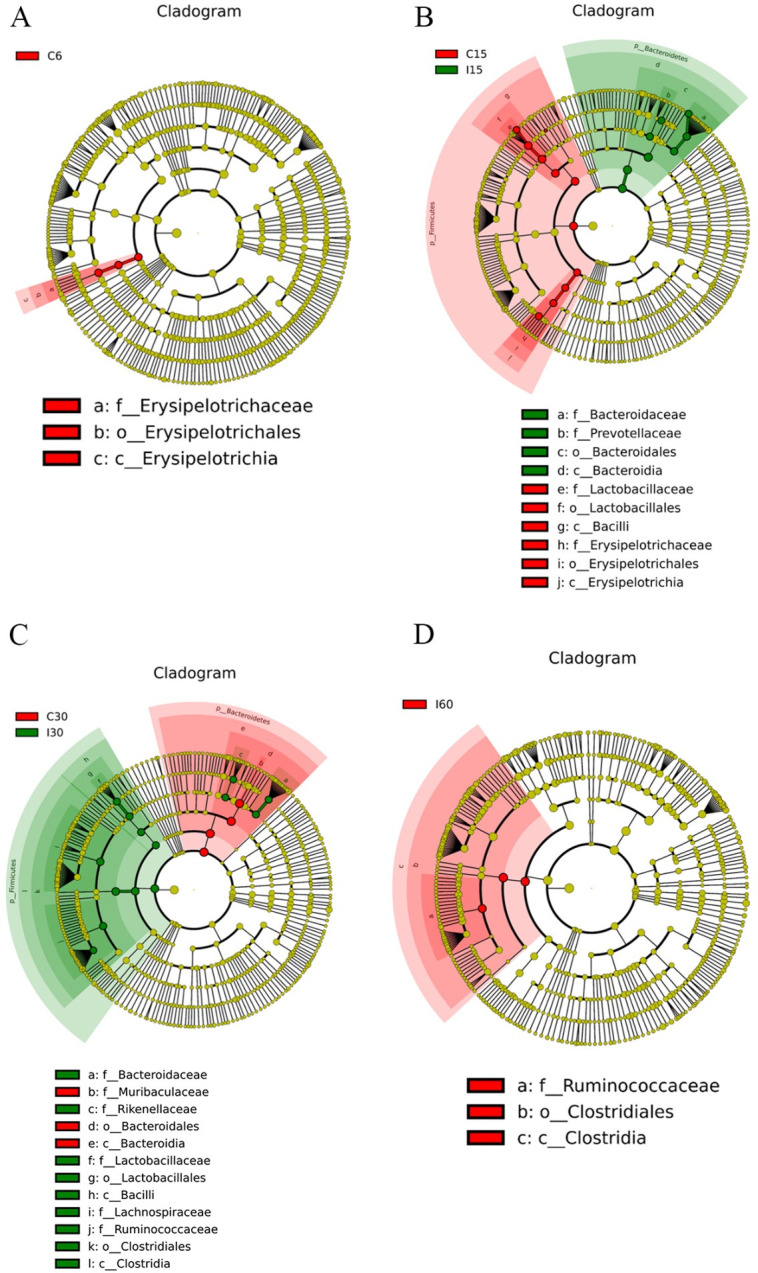
Bacterial Lineage Cladograms with Significantly Different Representation in Infected and Uninfected Groups. The lineages of bacteria on the trees were color-coded to indicate whether the taxon significantly differed between two groups at the genus level (red or green) or does not (yellow). (A) I6 vs. C6 group, (B) I15 vs. C15 group, (C) I30 vs. C30 group, (D) I60 vs. C60 group.

The quantitative microbiome analyses showed that the different amounts of *Bacteroidales* to Firmicutes in the infected and uninfected groups were statistically significant. Clearly, the major change was observed because of *T*. *spiralis* infection, with a linear discriminant analysis (LDA) effect size (log 10) of approximately 4. In the infected group, only *Erysipelotrichia* was reduced on day 6, *Bacteroidales*, *Bacteroides vulgtus*, *Alloprevotella* and *prevotellaceae* were more abundant on day 15, while *Lactobacillus*, *Bacilli*, *Erysipelotrichia*, *Duboisella*, *Jeotgalicoccus* and *lactobacillus animals* were reduced on day 15. In contrast, there was an increase in *Clostridia*, *lactobacillus*, *Bacilli*, *Alistipes*, *Ruminococcaceae*, *Lachnospiraceae*, and a decrease in *Rikenellaceae*, *Muribaculaceae* and *Bacteroidales* in infected mice on day 30 (Fig 5B–5E). Intriguingly, *Clostridia* and *Ruminococcaceae* continued to increase after day 60. Organizing these results into cladograms at the genus level provided an easily appreciated view of the microbiome shifts because of colonization with *T*. *spiralis* (Fig 6).

### Alteration of specific microbial taxa abundance is associated with immune characteristics during infection

**Fig 7:**
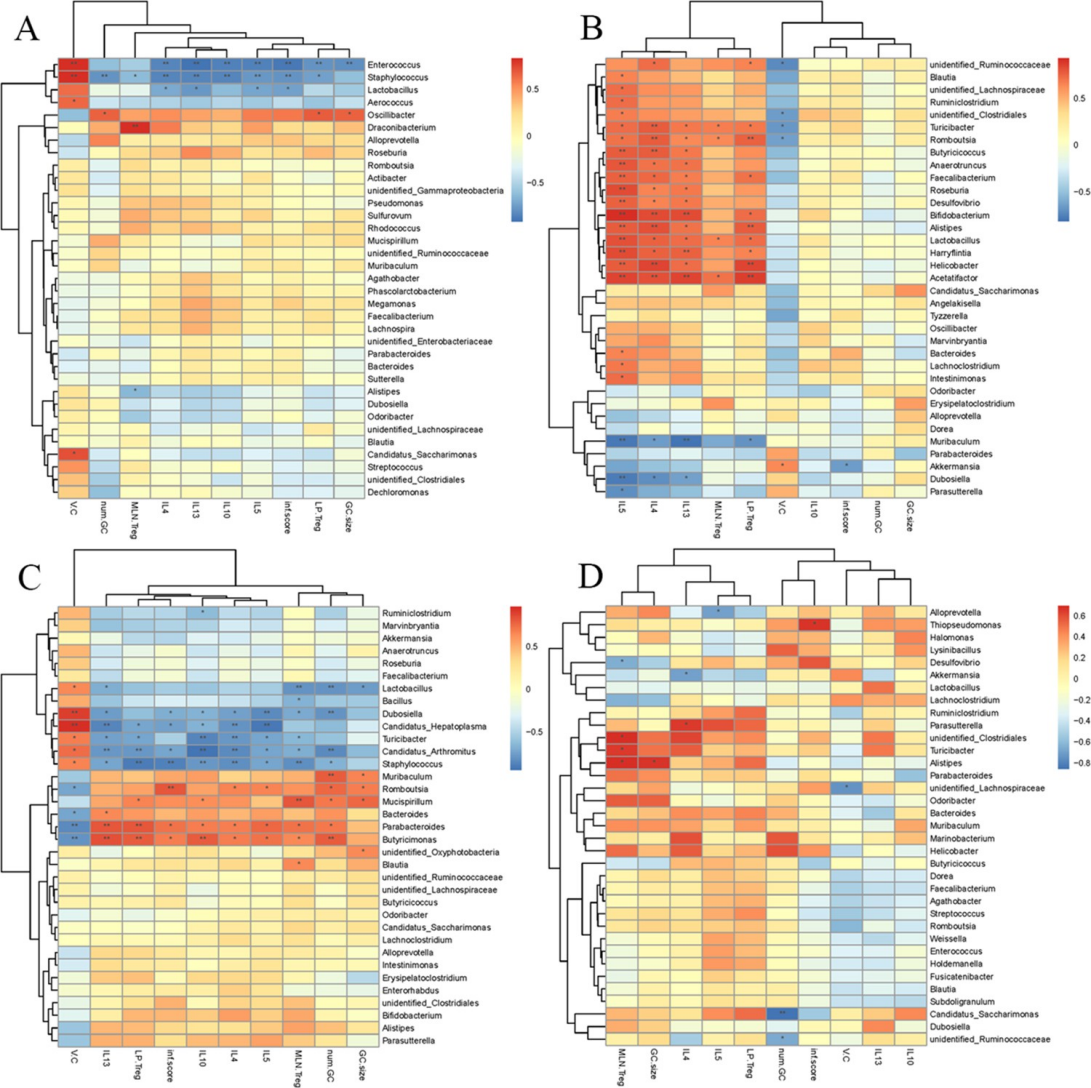
Microbial Structure is associated with Immune Characteristics. A heat map of the Spearman correlation coefficient score and statistical significance was calculated by Package Vegan from the R program. It showed the associations between variations in the gut microbiota and host immune characteristics during *T*. *spiralis* infection. (A) The associations were analyzed on day 6 after infection with *T*. *spiralis*. (B) The associations were analyzed on day 15 after infection with *T*. *spiralis*. (C) The associations were analyzed on day 30 after infection with *T*. *spiralis*. (D) The associations were analyzed on day 60 after infection with *T*. *spiralis*. V.C represented villus height/crypt depth, num. GC represented the number of goblet cells per villus, GC. size represented the size of goblet cells, inf. Score represented the inflammatory score. Each cell’s color represents the slope’s direction (red is positive, blue is negative). *P <0.05, **P <0.01.

We explored the associations between variations in the gut microbiota and host immune characteristics using Spearman correlation test analysis during infection. Here, we showed that the microbiota communities significantly correlated with the immunomodulation signatures (IL-4, IL-5, IL-13, IL-10, MLN Treg, and LP Treg) and duodenum pathological indicators (villus height/crypt depth, the number of goblet cells per villus, the size of goblet cells, and the inflammatory score). *Enterococcus* and *Staphylococcus* had significantly negative slopes for Th2/Treg immune response and duodenum tissue damage indicators, excluding villus height/crypt depth, showing that their abundance was lower in infected mice on day 6(Fig 7A). The highly prevalent genera *Lactobacillus*, *Bacillus*, *Dubosiella*, *Hepatoplasma*, *Turicibacter*, *Arthromitus*, and *Staphylococcus* also had significant negative slopes for Th2/Treg immune responses and duodenum tissue damage indicators as the newborn larvae circulation elicited systemic inflammation on day 15. For the genera *Butyricimonas*, *Parabacteroides*, *Bacteroides*, *Mucispirillum*, *Rombousia*, and *Muribaculum*, however, the slope of the Th2/Treg immune response and duodenum tissue damage indicators were strongly positive (Fig 7B). With the healing of tissue damage, bacteria such as *Ruminococcaceae*, *Blautia*, *Lachnospiraceae*, *Ruminiclostridium*, *Clostridiales*, *Turicibacter*, *Romboutsia*, *Butyricicoccus*, *Anaerotruncus*, *Faecalibacterium*, *Roseburia*, *Desulfovibrio*, *Bifidobacterium*, *Alistipes*, *Lactobacillus*, *Harryflintia*, *Helicobacter*, and *Acetatifactor* exhibited similar patterns of association with the Th2/Treg immune response, which indicated that these genera may be attempted to promote muscle larval colonization (Fig 7C). On day 60, we found that the genera *Alistipes*, *Clostridiales*, and *Turicibacter* had significant positive slopes for Treg (Fig 7D).

### Changes in serum metabolomic features in mice infected with *T*. *spiralis*

**Fig 8:**
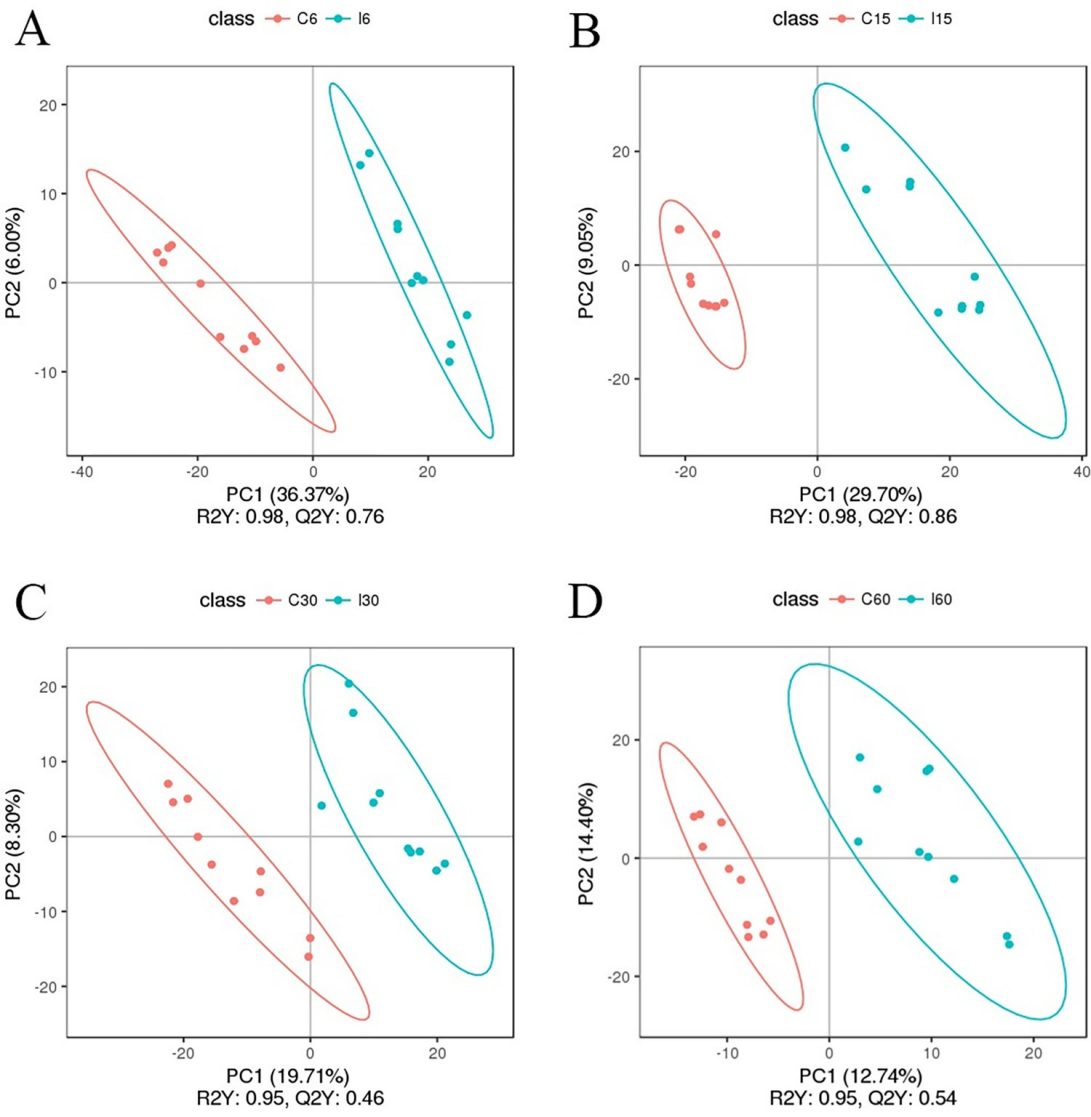
Partial Least Squares Discrimination Analysis (PLS-DA) Score Plots under Positive Ionic Mode. R2Y represented the interpretation rate of the model and Q2Y reflects model prediction. The closer R2Y and Q2 were to 1, the better the model stability and predictability. (A) I6 vs. C6 group. (B) I15 vs. C15 group. (C) I30 vs. C30 group. (D) I60 vs. C60 group.

Metabolomics was the simultaneous qualitative and quantitative analysis of all small molecule metabolites of an organism or cell during a specific physiological period. We identified the serum metabolites of the infected and uninfected mice at each time point and then generated untargeted metabolite profiles, which allowed them to identify and measure all of the metabolites in the samples, by Liquid Chromatograph Mass Spectrometer (LC-MS). We optimized the detection for both positive and negative ionic modes, which referred to the charge of the metabolites, considering the variable physicochemical properties of metabolites, such as molecular weight, polarity, solubility, and charge. In total, 3638 metabolites with positive and 2277 with negative ionic modes had been found. Because metabolome data had the characteristics of multi-dimension and high correlation among some variables, the analysis of those data required the use of multivariate statistical methods. Partial least squares discriminant analysis (PLS-DA), a commonly used method in metabolome analysis, could generally reflect the overall metabolic difference between the samples of each group and the variation degree within the group. Based on the PLS-DA models of metabolite profiling data, we found that the serum metabolites significantly differed between infected and control mice (Figs 8 and S1). The significant difference in metabolites suggested that the parasite infection impacted mouse metabolism.

**Fig 9:**
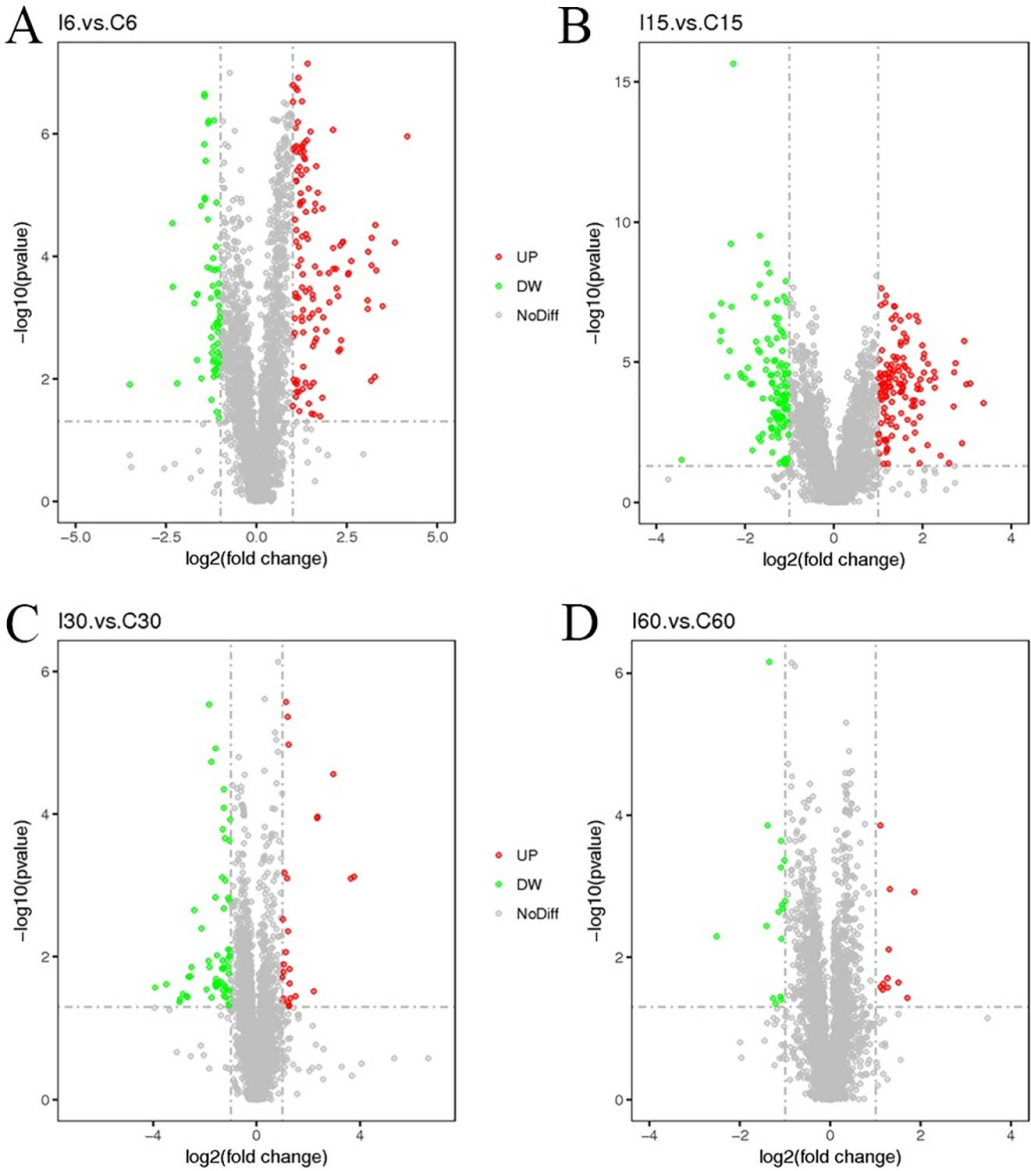
Volcano Plot under Positive Ionic Mode. Each point represented a metabolite. The horizontal coordinate represented the multiple change of the relative substances in the group (taking the logarithm of base 2). The vertical coordinate represented the *P* value of T test (taking the logarithm of base 10). The size of the scatter point represented the VIP value of the PLS-DA model. The larger the scatter point, the larger the VIP value. Significantly up-regulated metabolites were shown in red, significantly down-regulated metabolites were shown in blue, and non-significantly differentiated metabolites were shown in gray. (A) I6 vs. C6 group. (B) I15 vs. C15 group. (C) I30 vs. C30 group. (D) I60 vs. C60 group.

From the PLS-DA models, we used the Variable Importance in the Projection (VIP), combined with the *P*-value of T-test to find metabolites of differential expression. The threshold was set as VIP>1, Fold Change (FC) > 2.0 or FC < 0.5 and P value<0.05. We identified differentially produced compounds that included 184 metabolites in positive ionic mode and 176 metabolites in negative ionic mode on day 6, 257 metabolites in positive ionic mode and 272 metabolites in negative ionic mode on day 15, 88 metabolites in positive ionic mode and 131 metabolites in negative ionic mode on day 30, and 27 metabolites in positive ionic mode and 48 metabolites in negative ionic mode on day 60 (Table 1). We visualized the results for differential metabolites in the form of a volcano plot. On day 6 following infection, 124 metabolites were up-regulated under the positive ionic mode, 60 were down-regulated, 88 were up-regulated, and 88 were down-regulated under the negative ionic mode as shown in the volcano plot. The number of different metabolites peaked on day 15, 136 metabolites were significantly up-regulated, 121 metabolites were significantly down-regulated under the positive ionic mode, 160 metabolites were significantly up-regulated, and 112 metabolites were significantly down-regulated under the negative ionic mode after infection. On day 30, 26 metabolites were significantly up, 62 were significantly down-regulated under positive ionic mode, 62 were significantly up-regulated, and 69 were significantly down-regulated under negative ionic mode. On day 60, only 11 metabolites were significantly up-regulated, and 16 were significantly down-regulated, under positive ionic mode. In contrast, 9 metabolites were significantly up-regulated, and 39 were significantly down-regulated. On day 60, only 11 metabolites were significantly up-regulated, and 16 were significantly down-regulated under positive ionic mode. In contrast, 9 metabolites were significantly up-regulated, 39 were significantly down-regulated (Figs 9 and S2). There was a significant difference between the infected group and the corresponding uninfected group at each time point, according to heat maps generated using the all-affected metabolites (S3 Fig).

**Table 1:** The number of different metabolites at each time point*.

Compared Samples	Num. of Total Ident.	Num. of Total Sig.	Num. of Sig.Up	Num. of Sig.down
**I6.vs.C6_pos**	**3638**	**184**	**124**	**60**
**I15.vs.C15_pos**	**3638**	**257**	**136**	**121**
**I30.vs.C30_pos**	**3638**	**88**	**26**	**62**
**I60.vs.C60_pos**	**3638**	**27**	**11**	**16**
**I6.vs.C6_neg**	**2277**	**176**	**88**	**88**
**I15.vs.C15_neg**	**2277**	**272**	**160**	**112**
**I30.vs.C30_neg**	**2277**	**131**	**62**	**69**
**I60.vs.C60_neg**	**2277**	**48**	**9**	**39**

*The differences were defined as VIP> 1.0, FC > 2.0 or FC < 0.5 and *P*< 0.05.

**Fig 10:**
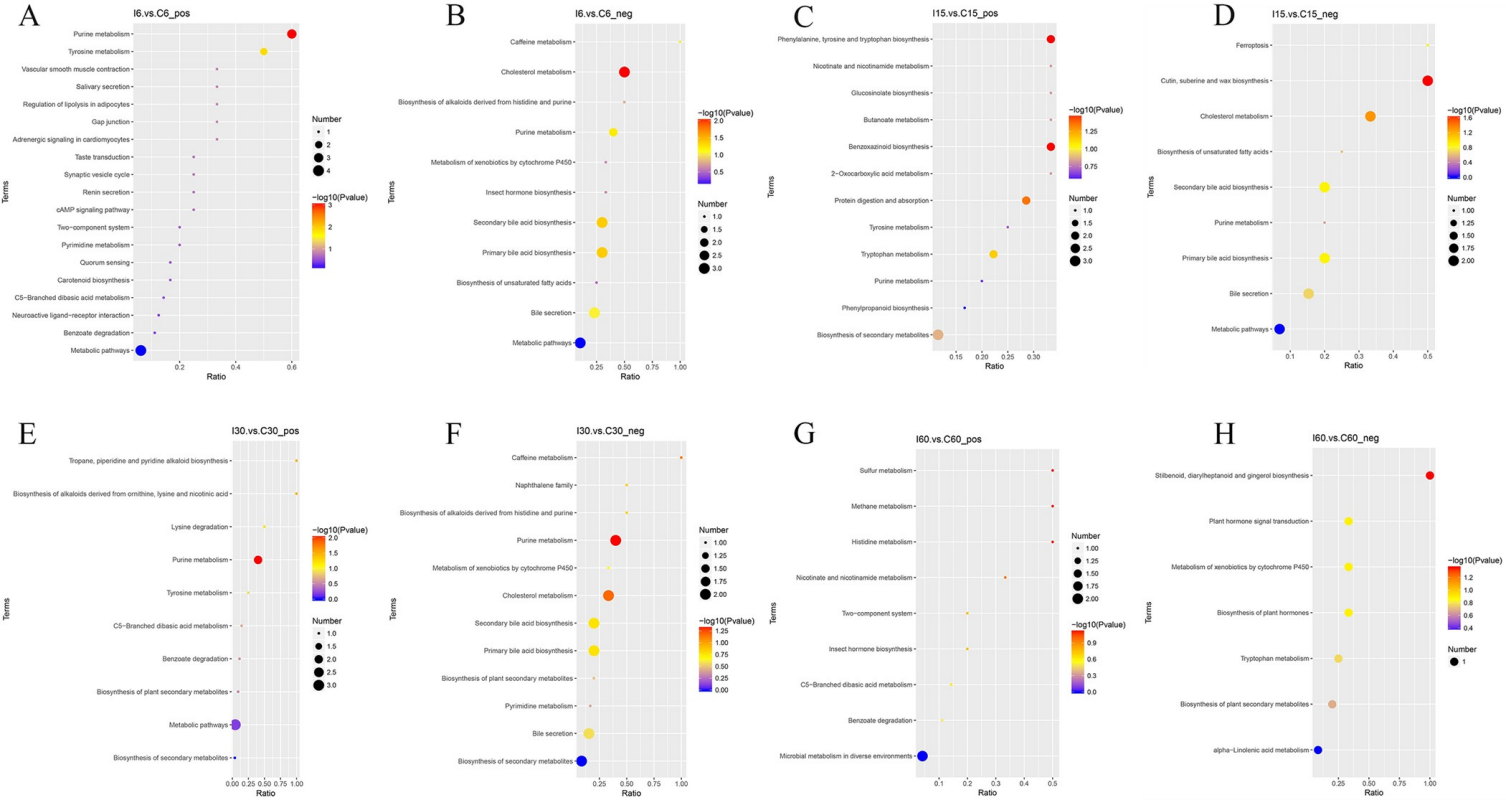
Analyses of KEGG Metabolic Pathways in Infected and Control Mice. DAVID was used to identify the altered metabolic pathways by *T*. *spiralis* infection on days 6 (A-B), 15(C-D), 30(E-F), and 60(G-H). A redder circle color indicated more-significant changes in the metabolites in the corresponding pathway, while the size of each circle corresponded to the pathway impact score (larger circles reflected higher centrality of the metabolites involved).

Kyoto Encyclopedia of Genes and Genomes (KEGG) was a database for systematic analysis of gene function and genomic information, which helped researchers to communicate genes and their expression information as a whole network. KEGG was a powerful tool for metabolism analysis and metabolic network research *in vivo*. DAVID v6.7 bioinformatic resources were used to predict the functions of *T*. *spiralis* infection-altered metabolic profiles and to identify potential therapeutic targets. Five and three pathways were significantly affected on days 6, 15, and 30, respectively (*p*<0.05 and enrichment ≥1). On day 60, there were no noticeable differences in KEGG pathways (Fig 10). On day 6, KEGG pathway analysis revealed that primary and secondary bile acid biosynthesis (Map ID: 00120 and 00121) were the most significantly down-regulated metabolic pathways, while purine metabolism (Map ID: 00230) and tyrosine metabolism (Map ID: 00350) were the most significantly up-regulated metabolic pathways. By day 15, cholesterol metabolism (Map ID: 04979) was still down-regulated. On day 15, there was also an increase in protein digestion and absorption (Map ID: 04974) and phenylalanine, tyrosine, and tryptophan biosynthesis (Map ID: 00400). Meanwhile, there was a corresponding decrease in hyperimmune metabolic pathways, including purine metabolism (Map ID: 00230), tropane, piperidine, and pyridine alkaloid biosynthesis (Map ID: 00960), and the biosynthesis of alkaloids derived from ornithine, lysine, and nicotinic acid (Map ID: 01064) on day 30, which returned to homeostasis in the encapsulated phase.

**Fig 11:**
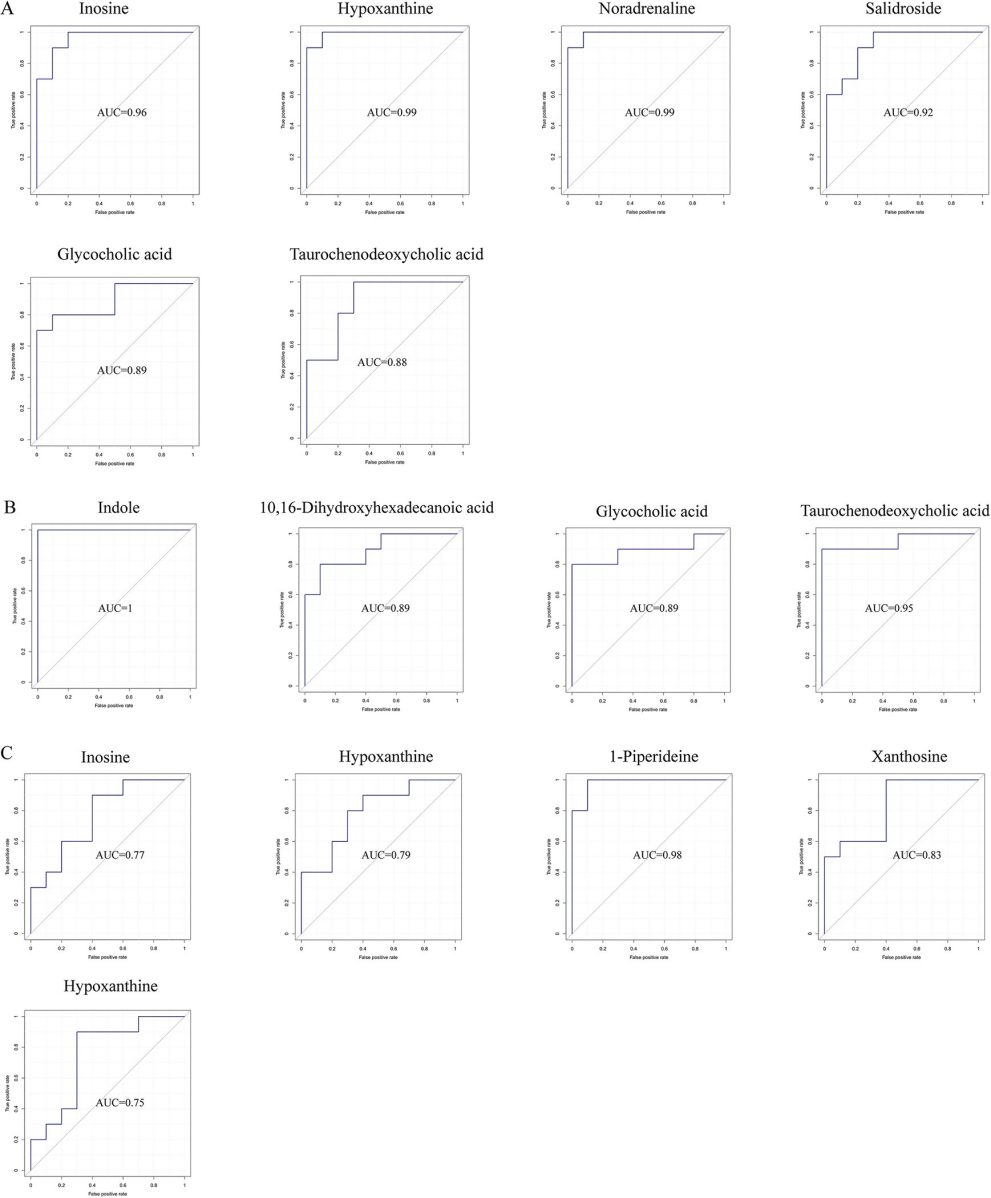
ROC Analyses of Potential Biomarkers for *T*. *spiralis* Infection. The score was higher than the cutoff (0.7). ROC analyses were performed to measure the sensitivities and specificities of these metabolites from corresponding pathways for *T*. *spiralis* infection on days 6 (A), 15(B), and 30(C).

Receiver operator characteristic (ROC) was drawn with the true positive rate (sensitivity) as the ordinate and the false positive rate (1-specificity) as the abscissa. The obtained differential metabolites were evaluated by the ROC curve for potential biomarkers. The Area below the ROC Curve was known as the Area Under Curve (AUC), and the AUC was used to assess the sensitivity and specificity of the biomarker for predicting the occurrence of an event. The closer the AUC value is to 1, the higher the accuracy of the prediction. ROC analysis revealed up-regulated inosine, hypoxanthine, noradrenaline, salidroside, and indole and down-regulated glycocholic, taurochenodeoxycholic acid, and 10,16-dihydroxyhexadecanoic acid as having the highest sensitivity and specificity to detect *T*. *spiralis* infection on days 6 and 15 (Fig 11A and 11B). In contrast, on day 30, the down-regulated inosine, hypoxanthine, 1-piperidine, and xanthosine had the highest sensitivity and specificity for detecting *T*. *spiralis* infection (Fig 11C).

### Correlation analysis reveals the relationship between serum metabolites and immune characteristics during infection

**Fig 12:**
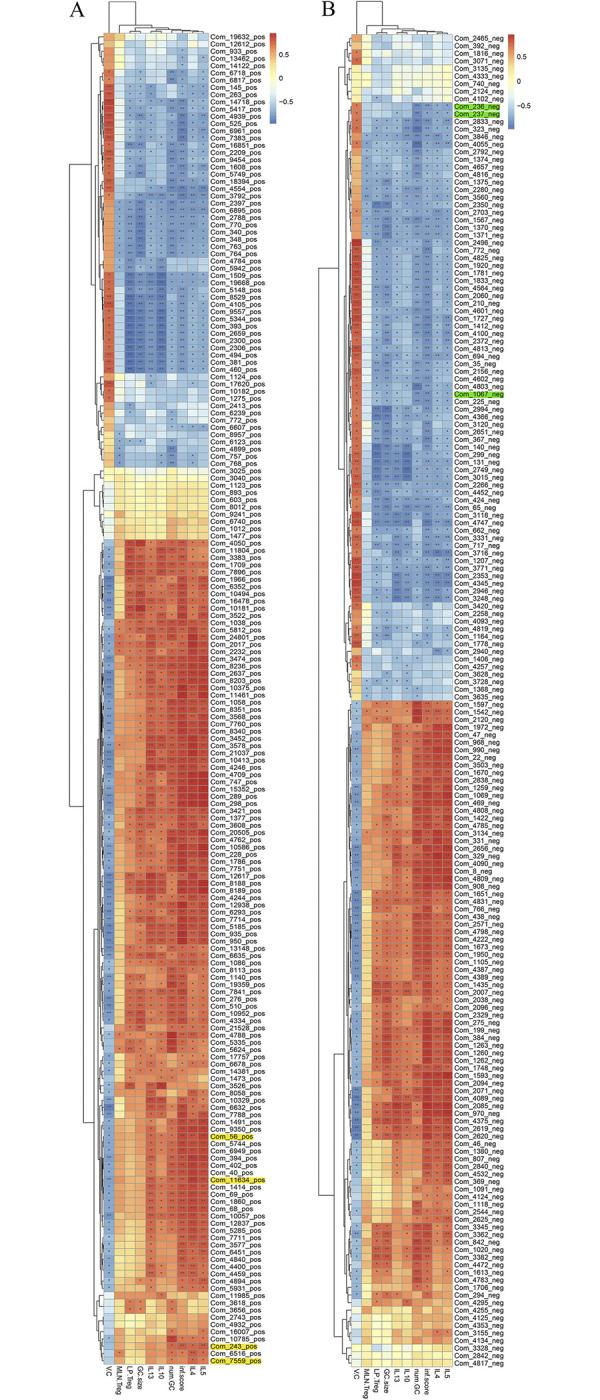
Metabolite Profiles associated with Immune Characteristics. A heat map of the Spearman correlation coefficient score and statistical significance were calculated by Package Vegan from the R program. It showed the associations between serum metabolites and host immune characteristics during *T*. *spiralis* infection. (A) The serum metabolites were analyzed in positive ionic mode. (B) The serum metabolites were analyzed in negative ionic mode. V.C represented villus height/crypt depth, num. GC represented the number of goblet cells per villus, GC. size represented the size of goblet cells, inf. Score represented the inflammatory score. The color of each cell represented the direction of the slope (red was positive, blue was negative). *P <0.05, **P <0.01, ***P <0.001.

We investigated the associations between serum metabolites and host immune characteristics using Spearman correlation test analysis during infection to elucidate the correlation between serum metabolites and immune characteristics during infection and further explore whether metabolism supports the outline of the host immune landscape (Fig 12A and 12B). With a *P* value of 0.05, we found a significant correlation between immunomodulation signatures (IL-4, IL-5, IL-13, IL-10, MLN Treg, and LP Treg) and duodenum pathological indicators (villus height/crypt depth, the number of goblet cells per villus, goblet cell size, and inflammatory score). The Th2/Treg immune response and duodenum tissue damage indicators for the potential biomarkers inosine, hypoxanthine, noradrenaline, salidroside, and indole all had significant positive slopes on days 6 and 15. While glycocholic, taurochenodeoxycholic acid, and 10,16-Dihydroxyhexadecanoic acid had significant negative slopes for Th2/Treg immune response and duodenum tissue damage indicators on days 6 and 15. In contrast, inosine, hypoxanthine, 1-piperidine, and xanthosine exhibited opposite patterns of association with the Th2/Treg immune response on day 30.

## Discussion

Helminths have immunomodulatory mechanisms that can help trigger Th2 and Treg responses, and helminth infection can influence the Th1 and Th17 responses [39]. Intestinal helminths have been shown to cause major changes in the structure and function of the host gut microbiota [20,40–42]. Furthermore, the gut microbiome and metabolites are intricately connected, and alterations in the gut microbiota profoundly influence downstream metabolic pathways [43]. The evidence for a relationship between the gut microbiota and the metabolome is robust and promising [44–49]. The host immune system is also modulated by changes to the gut microbiota and metabolites, which affect the host immune regulatory network and, ultimately, helminth infection and parasitism [50,51]. The gut microbiota is important in determining host permissiveness for parasites [22,52]. The parasite manipulates host metabolism via insulin signaling and the gut microbiome [53,54]. Determining the effects of helminth infection-induced immune regulation characteristics on the intestinal microbiota and metabolic pathways in mice, as well as the connections between helminth-induced immune regulation characteristics and changes in mouse intestinal microbiota and metabolic pathways, are therefore crucial. It will contribute to a better understanding of the host-parasite interaction, define "worm therapy," clarify its mechanism and principle, and give a theoretical framework for the use and development of worm therapy for intestinal inflammatory diseases.

The interaction between helminth-induced immune regulation, gut microbiota, and metabolic pathways has yet to be investigated. To evaluate changes in immunological features, 10 *T*. *spiralis*-infected mice were collected at different time points (day 0, day 6, day 15, day 30, and day 60). To investigate the impact of *Trichinella* colonization on gut microbiota and serum metabolites and to study changes in gut microbiota or serum metabolites and immunological signatures, gut microbiota sequencing will be achieved in stool samples, and metabolomic characterization of serum samples will be performed. Understanding how helminth infection affects the gut microbiota or metabolic pathways by modifying immunity may help helminths cure intestinal diseases.

We investigated the changes in *Trichinella*’s physiological and immune characteristics at different developmental stages of mouse infection. Each C57BL/6 mouse was gavage with 400 *Trichinella* muscle larvae. The worm burden fluctuated according to developmental stage, and the body weight was always lower than normal mice. We also conducted duodenal tissue sections and active inflammatory parameters of the host at each developmental stage of *T*. *spiralis*. We found that the host’s intestinal lesions were more severe in the early intestinal stage of *Trichinella* infection (days 6 and 15). *Trichinella* causes damage to the host’s gastrointestinal tract, and changes in damage to the intestinal tract follow *Trichinella*’s developmental cycle. On day 6 of infection, the study found elevated IL-4, IL-5, IL-13, and IL-10 levels in infected mice. These levels remained elevated until day 30 and returned to normal by day 60. These findings aligned with the findings of previous studies which found the early immune response in the intestine during *T*. *spiralis* infection was mediated by cytokines and intestinal mucosal immune cells, including dendritic cells (DCs), eosinophils, goblet cells, and mast cells. Th2 cells produced cytokines that led to changes in these immune cells during *T*. *spiralis* infection. DCs were important antigen-presenting cells in the intestine and played a key role in regulating the mucosal immune response. *T*. *spiralis* infection could induce a strong Th2 response, which contributed to worm expulsion and goblet cell hyperplasia. Mastocytosis in the intestine during *T*. *spiralis* infection depended on the interplay between stem cell factor (SCF) and Th2-derived cytokines, such as IL-3, IL-4, and IL-9. These immune responses contribute to the host’s defense against *T*. *spiralis* infection [55–59]. Th1 cell cytokines IL-2 and IFN-γ showed a delayed increase, with levels only rising on day 15, in contrast to the Th2 cell cytokines. On day 6, we also noticed that IL-17A levels declined while IL-6 levels surged. *T*. *spiralis* infection had been shown to increase the recruitment of Treg cells, which could suppress IL-17A production and reduce inflammation [60].

Helminth infection can induce Th2 and Treg immune responses, which means that Th2 cytokines and Treg are significantly increased in mesenteric lymph nodes early in *Trichinella* infection and gradually return to normal levels over time. This is also consistent with *Trichinella*’s developmental cycle. T-helper type-2 (Th2) cells are activated during the complicated immune-mediated process of *T*. *spiralis* worm expulsion from the small intestine. *T*. *spiralis* larvae invade the intestine, and Th2-related cytokines increase immediately. IL-4 and IL-13 levels peak before the formation of full-muscle larvae [61]. This may be because of the early stage of *T*. *spiralis* infection, which damages the intestinal epithelium and the underlying tissue layer, which cause the body’s immune system to respond to regulate the immune system. Additionally, *T*. *spiralis* infection increased the number of small intestine mucosal immune cells and provided further evidence that the intestinal mucosal immune system of infected mice was induced towards a mixed Th1/Th2 phenotype, with a predominance of Th2 responses in the early stage of infection [62]. However, these finding were inconsistent with the findings of other studies, which suggested a different pattern of immune response during *T*. *spiralis* infection. According to these studies, the initial response during the intestinal stage was dominated by Th1 cytokines such as IFN-γ and IL-12, which were involved in activating immune cells to fight the parasite. However, during the muscle stage of infection, there was a shift towards a Th2 response, characterized by the production of cytokines such as IL-4, IL-10, and IL-13, which were involved in regulating inflammation and tissue repair [63,64]. Similarly, the first response during the intestinal phase was a moderate Th1 response, which was subsequently shifted to a powerful Th2 response. Th2 immunity was also a powerful muscle defense mechanism, and the regulatory arm of immunity was engaged to protect both the parasite and the host [65]. Notably, levels of Th1-type cytokines IL-2 and IFN-γ were elevated during the migratory stage of newborn larvae (day 15), which could be attributed to macrophage stimulation and induction the DCs maturation during larval migration. TNF-α and IFN-γ production during *T*. *spiralis* infection may result from macrophage activation [66]. Many studies have shown that Th1 cytokines, such as IL-12, INF-γ, IL-1β, and TNF-α are much more abundant in the early stages of intestinal infection by *T*. *spiralis* [63,67–69]. Inflammatory mediators, such as prostaglandin E2 (PGE2) and nitric oxide (NO), as well as the production of Th1 cytokines, such as, IL-12, INF-γ, IL-1β and TNF-α, were found to be produced in greater amounts during the intestinal phase of the *T*. *spiralis* infection, which was linked to an increased number of eosinophils and the development of intestinal pathology [64,70]. This suggested that *T*. *spiralis* infection induced an increase of small intestine mucosal immune cells and a mixed Th1/Th2 phenotype with a predominance of Th2 response at the early stage of infection.

Infection with *T*. *spiralis* induced a Type-2 immune response, leading to helminth expulsion. However, Treg cell levels in the mesenteric lymph nodes were elevated in infected mice, with a peak on day 15 and a return to normal levels by day 60. The increase in the frequency of Tregs helped the helminth persist. The number of Treg in the colonic lamina propria remained high in late-stage *T*. *spiralis* infection, showing that other factors in the gut regulate the immune system in response to *T*. *spiralis* damage to the intestinal lamina propria and epithelium. Tregs were vital in maintaining intestinal immunological homeostasis, particularly in the colon, raising the possibility that bacterially driven modulation of host immune responses might contribute to the altered worm burdens [71]. Helminth infections had been shown to increase the amount of colonic Tregs and Foxp3 expression, both of which were critical in maintaining the immunological balance in the colon [14,72]. On the same vein, *T*. *spiralis* infection can induce a polarized Th2 immune response, characterized by producing Th2 cytokines (IL-4, IL-10) and developing Treg cells [73,74].

Through 16S rRNA gene-based sequencing, this study examined the influence of *Trichinella* infection on gut microbiota throughout chronic infection. We have determined that helminth infection in female C57BL/6 considerably impacted the host microbiota. At days 6 and 60, there were no significant differences in the α-diversity of the gut microbiota between the infected and uninfected groups. Compared to the uninfected group, the infected group showed a higher α-diversity using the Simpson index on day 15, higher observed species values, and a higher α-diversity using the Shannon index on day 30. Our findings were inconsistent with a previous study that found a significant decrease in α-diversity on day 28 because of infection, which became more apparent by day 41 [75]. The β diversity of the microbiota differed significantly between the infection and uninfected groups at days 30 and 60. The difference in the β-diversity increased by day 41, showed by both DGGE and sequencing, confirmed the impact seen in our study assessing changes in cecal microbiota [75]. Several studies had found changes in the microbiota in the colons of pigs on day 21, which corresponded to the larval stages [76,77]. Helminth infection had resulted in a slight increase in diversity, with people carrying a range of helminth infections, including *Necator americanus* and *Ascaris* spp., making it difficult to determine cause and effect [78]. However, no significant alterations in microbiota were reported in a human investigation when *N*. *americanus* was the only soil-transmitted helminth [79]. There might be a variety of explanations for the observed variations between these experiments, and it might be partly because various parasites were studied, including mixed infections. Similarly, changes in sample preparation and DNA amplification for sequencing, subsequent sequence analysis, and reference database selection might account for some of these variances.

*Bacteroidetes* were the most abundant bacterial phylum, followed by *Firmicutes*, *Proteobacteria*, *Actinobacteria*, *Deferribacteres*, *Chloroflexi*, *Verrucomicrobia*, *Acidobacteria*, and *Fusobacteria*. The most obvious changes were the relatively higher numbers of *Bacteroidales* and lower numbers of *Firmicutes* on days 6 and 15, which were reversed on days 30 and 60. These findings coincided with the result that infection with *T*. *spiralis* decreased the ratio of *Firmicutes* to *Bacteroidetes* in the gut, which helps restore the previously increased ratio observed in high-fat diet-fed mice [80]. *Bacteroidetes*, in particular, accounted for over 66% of sequences in the infection group on day 15, compared to only about 43% in the uninfected group. At day 30, colonization by *T*. *spiralis* was associated with a 2.4-fold increase in the relative number of *Firmicutes* sequences. Additionally, colonization by *T*. *spiralis* had similar effects on *Proteobacteria*, which decreased from a 1.7-fold increase on day 15 to a 2.7-fold decrease on day 60. Our findings on how infection affected microbiota were consistent with previous research that found helminth infection to significantly impact the host microbiota in C57BL/6 mice [75]. During intestinal helminth infection, a complex relationship emerged between the worm, the host immune system, and the intestinal microbiome. The intestinal microbiome interacted with the host immune system to control helminth infection by shaping microbial communities and activating host immunoregulatory pathways [81]. The presence of a complex bacterial microbiota provides the host with resistance against intestinal helminths by regulating intestinal motility [82].

Changes in the gut microbiome and vice versa might affect the immune response to *T*. *spiralis* infection. Infection with *T*. *spiralis* induced a switch from elevated *Bacteroidales* in the parenteral phase to Treg-*associated Clostridiales*, *Ruminococcaceae*, *Lachnospiraceae* and *Bacteroides* in the encapsulated phase. *T*. *spiralis* induced the expansion of colon lamina propria Tregs, which might occur through indirect mechanisms involving the intestinal microbiome. In this respect, *Bacteroidetes* were gastrointestinal bacteria that broke down carbohydrates [83]. The anaerobic breakdown of carbohydrates released short-chain fatty acids (SCFAs), which were easily absorbed by the host and enable the host to obtain energy from sources that would otherwise be inaccessible by *Bacteroidetes* fermentation. Variations in the SCFA levels in the intestine could alter the lumen’s pH and impact the composition of the microbiota present [84]. The mice used in the study were given a diet rich in plant-based fiber that includes feed manufactured from wheat, barley, soy, and maize. The intestinal immune system had recently been shown to be significantly influenced by bacterially produced SCFA, which might also directly trigger CD4^+^ Foxp3^+^ Treg cells in the colon [85]. Helminth-regulation of inflammatory disease, demonstrating a crucial function for bacteria-derived SCFAs acting through ffar2 in *H*. *diminuta*-amelioration of colitis, the critical necessity of IL-10 that could up-regulate the expression of SCFA transporters/receptors, and butyrate regulation of IL-10 receptor expression [43]. However, the decrease in IL-17 production is correlated with an increase in CD4^+^ Foxp3^+^ Treg cells in the germ-free colon. Animals were reconstituted with a complex and diversified microbiota that lacked the major phyla. *Bacteroidetes* can’t restore the proper immunological balance, demonstrating how various organisms could affect the pro- and anti-inflammatory responses in the gut [86]. Similarly, *T*. *spiralis* induced the expansion of colon lamina propria Tregs through the release of IL-33, which led to the expansion of GATA3^+^ Tregs. Loss of DRA from colonocytes triggers the release of IL-33, which drove a type 2 immune response and specifically expanded a subset of GATA3^+^ Tregs [87]. The underlying mechanisms of action involved an IL-17/TNF-alpha synergistic reaction, suppression of Th1 and Th2 responses, and an upregulation of the regulatory cytokines IL-10 and TGF-β1 [88]. The microflora was required to accumulate cytokine-producing CD4^+^ T cells in the colon lamina propria, including Th17 cells that produce IL-17 [89]. The exact mechanisms by which *T*. *spiralis* achieved inflammation modulation and induced the expansion of colon lamina propria Tregs have not been fully elucidated yet [90].

Immunomodulation signatures (IL-4, IL-5, IL-13, IL-10, MLN Treg, and LP Treg) and duodenal pathological indicators (villus height/crypt depth, the number of goblet cells per villus, the size of goblet cells, and the inflammatory score) were substantially connected with bacterial communities. Except for villus height/crypt depth, *Enterococcus* and *Staphylococcus* displayed substantially negative slopes for Th2/Treg immune response and duodenal tissue damage markers, showing that their abundance was decreased in infected animals at day 6. As the newborn larvae circulation elicited systemic inflammation at day 15, the highly prevalent genera *Lactobacillus*, *Bacillus*, *Dubosiella*, *Hepatoplasma*, *Turicibacter*, *Arthromitus*, and *Staphylococcus* also had significant negative slopes for Th2/Treg immune responses and duodenum tissue damage indicators, showing that this microbial community was decreased by inflammation. However, the Th2/Treg immune response slope and duodenum tissue damage indicators were strongly positive for the genera *Butyricimonas*, *Parabacteroides*, *Bacteroides*, *Mucispirillum*, *Rombousia*, and *Muribaculum*, showing that this microbial community is likely to be strongly stimulated by inflammation. *Turicibacter*, *Romboutsia*, *Butyricicoccus*, *Anaerotruncus*, *Faecalibacterium*, *Roseburia*, *Desulfovibrio*, *Bifidobacterium*, *Alistipes*, *Lactobacillus*, *Harryflintia*, *Helicobacter*, and *Acetatifactor* showed similar patterns of association with the Th2/Treg immune response with the healing of tissue damage, suggesting that these genera may be attempts to promote muscle larval colonization. We observed substantial positive slopes for Treg in the taxa *Alistipes*, *Clostridiales*, and *Turicibacter* at day 60. These results showed that immunological characteristics in the GALT were correlated with infection-related changes in the abundance of certain microbial species. During primary infection, Th2 and duodenal mucosal inflammation were mounted against intestinal helminths to eliminate luminally dwelling worms, and subsequent Treg expansion was required for effective parasite establishment. This was followed by a shift from elevated *Parabacteroides and Butyricimonas to elevated Lactobacillus*, *Turicibacter*, *Romboutsia*, *Faecalibacterium*, *Bifidobacterium*, *Alistipes*, *Harryflintia*, *Helicobacter*, and *Acetatifactor*, raising the possibility of bacterially driven modulation of host immune responses during infection. In line with these findings, mice treated with *Bifidobacteria infantis* in a model of pathogen-induced inflammation experienced a reduction in intestinal inflammation, increased CD4^+^CD25^+^Treg cells, and decreased Th1 and Th17 responses [91]. Treg cells are significantly reduced in number and functionality in germ-free animals [92,93]. There was limited information on the relationship between immunomodulation signatures, duodenal pathological indicators, and bacterial communities. To better understand their relationship, further research was needed to explore the mechanisms and interactions between immunomodulation signatures, duodenal pathological indicators, and bacterial communities.

A non-targeted LC-MS/MS-based serum metabolomic study was conducted in mice to detect *T*. *spiralis*-specific alterations in metabolites and metabolic pathways, as well as possible biomarkers of *Trichinella* infection. Metabolism was one of the key components of the host-parasite relationship, which allowed parasites to successfully invade and infect their hosts. This host-parasite metabolic relationship was dynamically regulated, reflecting distinct immune-metabolic demanded at each stage of parasite differentiation. Primary and secondary bile acid biosynthesis was the most significantly down-regulated metabolic pathways on day 6 of the study, and purine metabolism and tyrosine metabolism were the most up-regulated metabolic pathways. Cholesterol metabolism was still down-regulated on day 15. Similarly, *T*. *spiralis* infection in mice resulted in alterations in several metabolites. The levels of total cholesterol were significantly reduced in mice infected with *T*. *spiralis* [80]. On day 15, there was also an increase in protein digestion and absorption and phenylalanine, tyrosine, and tryptophan production. There were no discernible changes in the KEGG pathways on day 60. Studies had shown that cholesterol accumulation had contributed to inflammation recently, while bile acids and purines control inflammation [94,95]. The activation of the NLRP3 inflammasome might be influenced by various lipids and their derivatives. Bile acids derived from cholesterol degradation could induce the ubiquitination of NLRP3 via the upregulation of protein kinase A (PKA) and inhibit its activation [96,97]. Most acute-phase proteins were rich in aromatic amino acids (phenylalanine, tyrosine, and tryptophan) during inflammation [98]. When glycolysis was inhibited, amino acids such as alanine or serine could also be converted to pyruvate, which was thought to affect NLRP3 activation [97]. Therefore, during the early stages of *T*. *spiralis* infection, these changes in metabolic pathways might develop a protective immune system for the host, followed by a balanced immune response to ensure muscle larvae colonization. On day 30, there was a corresponding decrease in hyperimmune metabolic pathways such as purine metabolism, tropane, piperidine, and pyridine alkaloid biosynthesis, and alkaloid biosynthesis from ornithine, lysine, and nicotinic acid. During the encapsulated phase, this decreased activity returned to homeostasis. These findings suggested that *T*. *spiralis* switched from an immune activation to an immune suppression metabolic pathway to colonize the host. On days 6 and 15, ROC analysis revealed that inosine, hypoxanthine, noradrenaline, salidroside, and indole had the highest sensitivity and specificity to detect *T*. *spiralis* infection, while glycocholic, taurochenodeoxycholic acid, and 10,16-dihydroxyhexadecanoic acid had the lowest. These findings are comparable to other findings [99].

However, the down-regulated metabolites inosine, hypoxanthine, 1-piperidine, and xanthosine had the highest sensitivity and specificity for identifying *T*. *spiralis* infection on day 30. This suggested that these metabolites may serve as diagnostic biomarkers of *T*. *spiralis* infection in mice during distinct periods. The putative biomarkers inosine, hypoxanthine, noradrenaline, salidroside, and indole all showed significant positive slopes on days 6 and 15, showing that these metabolites increased as inflammation increased. However, on days 6 and 15, glycocholic, taurochenodeoxycholic acid, and 10,16-Dihydroxyhexadecanoic acid had significant negative slopes for Th2/Treg immune response and duodenum tissue damage indicators, indicating that these metabolites were decreased by inflammation. Inosine, hypoxanthine, 1-piperidine, and xanthosine; however, exhibited opposite patterns of association with the Th2/Treg immune response at day 30, implying that lowering these metabolites may be proposed to promote muscle larvae colonization. These findings raise the possibility that metabolites influence host immune responses during infection. Th2 and duodenum mucosal inflammation during primary infection are mounted against intestinal helminths to expel luminally dwelling worms with up-regulated inosine, hypoxanthine, noradrenaline, salidroside, indole and down-regulated glycocholic, taurochenodeoxycholic acid, and 10,16-Dihydroxyhexadecanoic acid. Subsequent Treg increases are essential for successful parasitism establishment with the healing of tissue damage, accompanied by down-regulated inosine, hypoxanthine, 1-piperidine, and xanthosine. Taurochenodeoxycholic acid (TCDCA) has anti-inflammatory and immune-regulating properties as a signaling molecule [100–102]. Xanthine and hypoxanthine are purine metabolites that induce inflammation by producing reactive oxygen species (ROS). Hesperidin supplementation increased ATP production and reduced oxidative stress in muscle in both in vitro and mouse models, while helminthic invasion may reduce the amount of muscle-protective compounds [99].

The experimental design had limitations, such as the difficulty of accounting for the effects of sampling on the microbiome (Fig 4A), immune responses, and metabolomics profiles (Fig 12). However, we addressed these limitations by standardizing sampling methods, using statistical methods to account for individual variation, conducting replicate experiments, using non-invasive sampling methods, and carefully monitoring the health of the mice. We used a cross-sectional design to assess histopathological and immunohistochemical changes at different stages of infection.

In summary, there are changes in the gut microbiota and metabolic activity, as well as parasite-induced immunomodulation. During the enteral and parenteral phases, the disrupted metabolisms adapt to infection stress and then return to homeostasis during the encapsulated phase. In the encapsulated phase, there was a change from an abundance of *Bacteroides* to an abundance of probiotic *Lactobacillus* and Treg-associated-*Clostridia*. Metabolite-based diagnostics based on potential biomarkers of *Trichinella* infection showed enormous promise for the future, allowing for rapid evaluation of animals and humans in the field. The present work focused on metabolites from the host; however, using metabolomics to investigate the microbiota, various phases of *Trichinella*, and integrate global metabolite levels with genomic, transcriptomic, and proteomic data to get systems-level knowledge of parasite metabolism and gene regulation might be of interest for the identification of novel targets for anthelmintic drugs and the development of metabolite-based diagnosis tools.

## Materials and methods

### Ethics statement

All animal procedures were approved by the Capital Medical University Institutional Animal Care and Use Committee (IACUC; Permit Numbers: AEEI-2015-183 and AEEI-2015-184). The principles of animal protection and care adhered to the NIH and IACUC guidelines for the Care and Use of Laboratory Animals.

### Parasites

The ISS 533 strain of *T*. *spiralis* was maintained by serial passage in female ICR mice in our laboratory. Each mouse was orally given 400 *T*. *spiralis* infective muscle larvae (ML). Adult worms were isolated from the small intestine of infected mice 5 days after infection (dpi). ML was isolated at 42 dpi from the muscles of infected mice according to modified pepsin–hydrochloric acid digestion. The muscle larvae (ML) were isolated from the muscles of infected mice at 42 dpi using a modified pepsin-hydrochloric acid digestion method, as previously described [103,104]. The ML was suspended in sterilized phosphate-buffered saline (PBS) and collected aseptically to prevent existing bacteria from influencing the gastrointestinal microbiota.

### Mice

Female C57BL/6 Wild type (WT) mice at 4–6 weeks old age were obtained from Capital Medical University’s Laboratory Animal Services Center (Beijing, China) and were kept in autoclaved cages in our closed container facility with a sealed top and force-filtered air. The mice had ad libitum access to sterilized feed and water and were handled in a biological safety hood with HEPA-filtered air. The mice were used two weeks after their arrival. For omics analysis, 80 WT mice were randomly assigned to infected (N = 40) and uninfected (N = 40) groups. TEN WT mice were used as the control group to collect the samples before *T*. *spiralis* infection on day 0. Foxp3^EGFP^ mice with a C57BL/6 background (B6.Cg-Foxp3^tm2Tch^/J; Shanghai Model Organisms Center Inc.) were bred in the same experimental facility as those described previously. To monitor the Treg cells during *T*. *spiralis* infection, 48 Foxp3^EGFP^ mice were randomly assigned to infected (N = 24) and uninfected (N = 24) groups. Infected mice were administered 400 pure ML suspended in sterile PBS orally, while controls received the same volume of PBS. To avoid cage effects, mice were co-housed for two weeks before treatment and infection. Each treatment group kept in the same cage for entire experiment.

### Sample collection

The worm burden was determined by counting them under a microscope. All animals were weighed on days 0, 6, 15, 30, and 60 dpi to track weight loss during infection. Ten animals from each group were euthanized to collect colonic contents and serum samples on days 0 (pre-infection), 6, 15, 30, and 60 dpi, respectively. Sera samples were obtained by centrifuging blood into 1.5 ml Eppendorf tubes at 3,000 g for 30 min. Sera were kept at -80°C until they were used. Feces or colonic contents were collected and frozen in liquid nitrogen for 10 min before being stored at -80°C for bacterial DNA extraction. Tissues from the duodenum and colon were removed and immediately fixed in 4% formaldehyde for histopathological and immunohistochemical staining.

### Hematoxylin and eosin (H&E) staining

After fixation and washing, duodenal tissues were dehydrated by soaking in alcohol and xylene, embedded in paraffin, and then sliced into 4-μm-thick sections, stained with H&E to visualize intestinal morphology [105]. A digital optical microscope was used to examine the slides (Olympus, Tokyo, Japan). Only full-length villi with adjacent crypts were measured for villus length and crypt depth, as described by Bejo [106]. The inflammation category of leukocytes is limited to histological scores: mild inflammation (score 1)-a detectable increase in lamina propria cellularity due to increased inflammation; moderate inflammation (score 2)-multifocal regions of increased lamina propria cellularity, with localized and distinct separation of the epithelial crypts from the tunica muscularis; severe inflammation (score 3)-similar to 2, but more extensive and diffuse, with inflammation separating most of the epithelium from the tunica muscularis.

### Immunofluorescence (IF) analysis of Foxp3^+^ cells in lamina propria

A frozen section procedure was performed, followed by the staining of CD4 with CD4-Cy3 antibody (Abcam brand, red light) and nuclei with DAPI (blue light). The Mouse Foxp3 had its own EGFP green fluorescence, and an automatic slide scanner was used for fluorescence mode scanning. The number of Treg cells can be estimated by observing them at 400× magnification.

### Cell culture and Treg staining

The mouse’s mesenteric lymph nodes (MLN) were used to prepare a single cell suspension in 1x lymphocyte separation medium, following the instructions from the manufacturer (Dakewe Biotech, Shenzhen, China). Subsequently, 2 × 10^6^ mesenteric lymph node cells/well in 1 ml of RPMI-1640 media containing 10% fetal bovine serum (FBS, Corning, USA), 100 U of penicillin/streptomycin/mL were cultured in 24-well plates at 37°C, 5% CO_2_ for 48 h in the presence of 2 μg/ml α-CD3/CD28(Biolegend, Europe, Uithoorn, the Netherlands). Culture supernatants were recovered after incubation for enzyme-linked immunosorbent assay (ELISA) by the corresponding ELISA kits for detecting IL-2, IL-4, IL-5, IL-6, IL-10, IL-13, IL-17A, TGF-β, and IFN-γ using the eBioscience ELISA Ready-SET-Go kit (eBioscience, San Diego, CA, USA) and Mouse ELISA KIT from Dakewe Biotech (Dakewe Biotech, Shenzhen, China) according to the manufacturer’s instructions. For FACS analysis of Tregs, the harvested MLN lymphocytes from each mouse were directly FcR antibody blocked and stained with a mouse regulatory T cell staining kit according to the manufacturer’s instructions (eBioscience, San Diego, CA, USA). The kit included stained antibodies against mouse CD4-FITC, mouse CD25-APC, and mouse Foxp3-PE. The surface marker staining was used: PerCP-labeled anti-CD3, FITC-labeled anti-CD4, and APC-labeled anti-CD25. After cells were washed, fixed, and permeabilized, intracellular staining was performed using PE-labeled anti-Foxp3 antibody and IgG2a mouse (negative control monoclonal antibody). The FlowJo flow (BD Biosciences, San Jose, CA, USA) cytometry analysis software was used to analyze the flow cytometry data.

### DNA extraction and 16S rRNA gene amplification, sequencing, and microbiota analysis

Total genomic DNA was extracted from fecal samples using the CTAB/SDS methods described in a previous study [107]. The concentration and purity of the DNA were determined using 1% agarose gel. The DNA concentration was diluted to 1 ng/μl using sterile water. The V4 hypervariable region of the 16S rRNA gene was amplified using specific primers (16S V4: 515F-806R) with the barcode [108]. All PCR reactions were performed using the Phusion High-Fidelity PCR Master (New England Biolabs). Following the manufacturer’s recommendations, sequencing libraries were generated using the Ion Plus Fragment Library Kit 48 rxns (Thermo Scientific). The library quality was assessed on the Qubit 2.0 Fluorometer (Thermo Scientific). The library was sequenced on an Ion S5 XL platform, and 400 bp/600 bp single-end reads were generated. According to Cutadapt, raw read quality filtering was performed under specific conditions to generate high-quality clean reads [109]. The reads were compared with the reference database [110] (Silva database, https://www.arb-silva.de/) using the UCHIME algorithm [111] to detect chimera sequences, and then the chimera sequences were removed [112]. The Clean reads were finally obtained. The Q20 (Quality score 20) for all samples was above 80 (S1 Data), meaning the probability of an incorrect base call was less than 1 in 100. This was considered to be a high-quality sequencing result.

### Microbiome data preprocessing and statistical analysis

Sequence analysis was performed by Uparse software (Uparse v7.0) [113]. Sequences with ≥97% similarity were assigned to the same OTUs. A representative sequence for each OTU was screened for further annotation. The Silva Database (Version 132) [110,114] for each representative sequence was used based on the Mothur algorithm to annotate taxonomic information. MUSCLE software (Version 3.8.31) was used for rapid multi sequence alignment to obtain the phylogenetic relationships of all OTUs representing sequences [115]. Finally, the data of each sample was homogenized based on the minimum amount of data in the sample. Then, we analyzed α diversity and β diversity on the homogenized data. The α diversity referred to the diversity within a sample, but β diversity referred to the diversity between samples. QIIME software (Version 1.7.0) was used to calculate Observed-OTUs, the Chao1 index (a measure of estimating the total number of microbial species), the Shannon index (a measure of considering the number of species and their relative abundance), the Simpson index (a measure of considering the number of species and their relative abundance, compared to the Shannon index, it was more influenced by uniformity), UniFrac distance (a measure of comparing whether there were significant differences in microbial communities in specific evolutionary lineages of samples)[116]. The Principal Coordinates Analysis was performed to obtain the principal coordinates and visualize the complex multidimensional data, which showed differences in community composition between different groups. PCoA analysis was displayed by the WGCNA package, stat packages, and ggplot2 package in R software (Version 2.15.3). The Unweighted Pair-group Method with Arithmetic Means (UPGMA) Clustering was performed as a hierarchical clustering method to interpret the distance matrix using average linkage and was conducted by QIIME software (Version1.7.0). LEfSe was used for the quantitative analysis of biomarkers within different groups. The linear discriminant analysis (LDA) effect size (LEfSe) method was used to identify significant enriched taxonomic units between the infected group and the uninfected group, which was performed by LEfSe software with a default LDA Score threshold of 4. Metastats analysis was conducted using the R software, employing permutation tests at various taxonomical levels (Phylum, Class, Order, Family, Genus, Species) to obtain p-values. The Benjamini and Hochberg False Discovery Rate method was then applied to correct the p-values and obtain q-values [117]. To analyze significantly different species between groups, the R software was used to perform group-wise T-tests and generate plots. The Spearman correlation coefficient score and statistical significance were calculated using the R vegan package, and the results reflect the correlation between abscissa and ordinate [118].

### Sample processing for metabolomics analysis

The samples were thawed and brought to room temperature. Metabolite extraction was performed as described, with a few slight changes [107]. Briefly, 100 μL of the liquid sample to be tested (ground in 0.1 mg tissue liquid nitrogen) placed in an EP tube, add 400 μL 80% methanol aqueous solution (4 times the volume of methanol), Vortex oscillation, stand at -20°C for 60 min, 14000 g, centrifuged at 4° C for 20 min. Take a certain amount of supernatant in 1.5 mL centrifuge tube, vacuum freeze—dried. The residue was dissolved in 100 μL of complex solvent, vortexed, centrifuged at 14000 g, 4° C for 15 min, and the supernatant was analyzed by LC-MS. An equal amount of supernatant was taken from each treated sample and mixed as QC sample. The blank sample was the blank matrix of the experimental sample, and the sample pretreatment process was the same as the experimental sample.

### Conditions for liquid chromatography

The sample extracts were examined using an LC-ESI-MS/MS (LC electrospray ionization MS/MS) system for nontargeted metabolomic analysis [39]. The following conditions applied to the analysis: Thermo Accucore HILIC column (2.6 μm, 3 mm × 100 mm); column temperature: 40°C; flow rate: 0.3 mL/min; mobile phase A was positive, containing 0.1% formic acid, 95% acetonitrile, and 10 mM ammonium acetate; mobile phase B was positive, containing 0.1% formic acid, 50% acetonitrile, and 10 mM ammonium acetate; mobile phase A (negative), was composed of 95% acetonitrile and 10 mM ammonium acetate (pH adjusted to 9.0); mobile phase B (negative) was composed of 50% acetonitrile and 10 mM ammonium acetate (pH adjusted to 9.0). The gradient program was as follows: 0% to 1 min, 98% A and 2% B; 1 to 17 min, 98% to 50% A and 2% to 50% B; 17.5 to 18 min, 50% to 98% A and 50% to 2% B; and 18 to 19 min, 98% A and 2% B. At level C, the ESI probe was reset. The following settings were made for the mass spectrometer: the spray voltage was set at 3.2 kV, the sheath gas flow rate was set at 35 arb, the aux gas flow rate was set at 10 arb, and the capillary temperature was set at 320°C. The mass spectrometer was equipped with positive and negative polarity. The raw data was then analyzed using the Thermo Compound Discoverer 3.0 software. The metabolites identified in the processed raw data of mass spectral peaks were searched for a matching fragmentation spectrum in the mzCloud database.

### Metabolomic data processing and analysis

The spectral data were analyzed using principal component analysis component analysis (PCA), latent structure projection, orthogonal (O)-PLS-DA, and discriminant analysis (PLS-DA)[119]. PCA was used to investigate any intrinsic similarities between samples. Then, PLS-DA was utilized to apply knowledge of infection status to maximize the separation of classes and biomarker recovery. O-PLS-DA [120] comprised an orthogonal data filter in PLS-DA and was used to improve the extraction of infection-related biomarkers by removing the impact of systematic variation independent of infection status [121]. Kyoto Encyclopedia of Genes and Genomes (KEGG) was a database for systematic analysis of gene function and genomic information [122]. Receiver operator characteristic (ROC) was drawn with the true positive rate (sensitivity) as the ordinate and the false positive rate (1-specificity) as the abscissa. The obtained differential metabolites were evaluated by the ROC curve for potential biomarkers [123].

The numerical data used in figures were included in **S2 Data**.

### Statistical analysis

The differences between groups were examined using one-way ANOVA with the Bonferroni correction. The data are shown as mean ± SEM; *p* < 0.05 is deemed statistically significant.

## Supporting information

S1 Data The quality of reads from sequencing demonstrated in QC stat. (XLSX)

S2 Data Excel spreadsheet containing, in separate sheets, the underlying numerical data and statistical analysis for Figs 1B, 1C, 1E, 1F, 1G, 2A, 2B, 3, 4B, 4C, 4D, 4E, 4F, 4G, 5A, 5B, 5C, 5D, 5E, 6A, 6B, 6C, 6D, 8A, 8B, 8C, 8D, 9A, 9B, 9C, 9D, 10A, 10B, 10C, 10D, 10E, 10F, 10G, 10H, 11A, 11B, 11C, S1A, S1B, S1C, S1D, S2A, S2B, S2C, and S2D. (XLSX)

S1 Fig Partial Least Squares Discrimination Analysis (PLS-DA) Score Plots under Negative Ionic Mode. R2Y represents the interpretation rate of the model and Q2Y reflects model prediction. The closer R2Y and Q2 are to 1, the better the model stability and predictability. (A) I6 vs. C6 group. (B) I15 vs. C15 group. (C) I30 vs. C30 group. (D) I60 vs. C60 group.
(TIF)

S2 Fig Volcano Plot under Negative Ionic Mode. Each point represents a metabolite. The horizontal coordinate represents the multiple change of the relative substances in the group (taking the logarithm of base 2). The vertical coordinate represents the *P* value of T test (taking the logarithm of base 10). The size of the scatter point represents the VIP value of the PLS-DA model. The larger the scatter point, the larger the VIP value. Significantly up-regulated metabolites are shown in red, significantly down-regulated metabolites are shown in blue, and non-significantly differentiated metabolites are shown in gray. (A) I6 vs. C6 group. (B) I15 vs. C15 group. (C) I30 vs. C30 group. (D) I60 vs. C60 group.
(TIF)

S3 Fig A Heat Map of the Metabolites Impacted by *T*. *spiralis* Infection in Mice. Each column represents a different sample and each row represents a different metabolite. Red indicates high expression metabolites, blue indicates low expression metabolites. Profiles of serum metabolites from positive (A-D) and negative ionic modes (E-H) were used to construct heat maps. Ten serum samples from infected mice and ten from control mice were analyzed at each time point. Red indicates that the metabolite had a higher expression level, and blue shows that the metabolite had a lower expression.
(PDF)
