# Supplementary figures and images for: Trichinella spiralis -induced immunomodulation signatures on gut microbiota and metabolic pathways in mice
Source: PLoS Pathog. 2024 Jan 2;20(1):e1011893. doi: 10.1371/journal.ppat.1011893 (PMC10786400; doi:10.1371/journal.ppat.1011893)

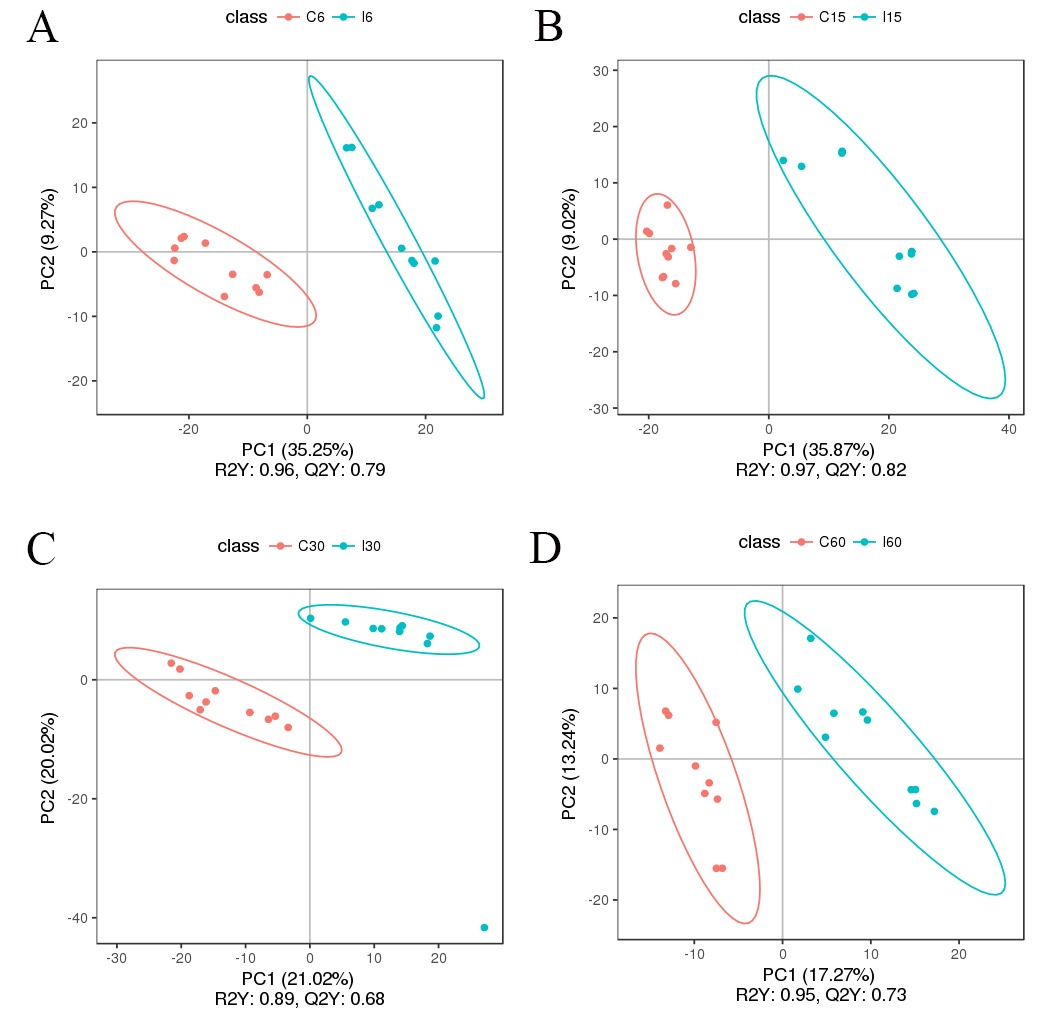

Supplement: S1 Fig — R2Y represents the interpretation rate of the model and Q2Y reflects model prediction. The closer R2Y and Q2 are to 1, the better the model stability and predictability. (A) I6 vs. C6 group. (B) I15 vs. C15 group. (C) I30 vs. C30 group. (D) I60 vs. C60 group. (TIF) [file ppat.1011893.s003.tif]

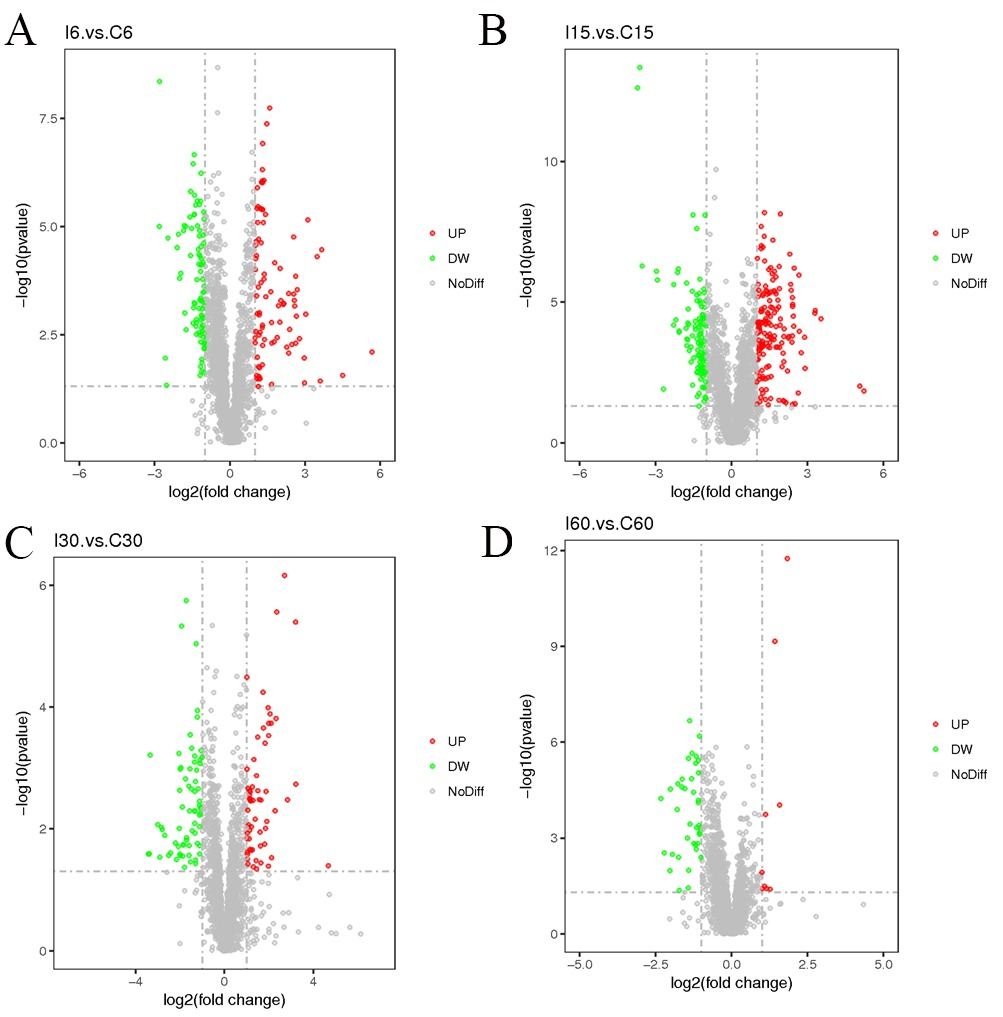

Supplement: S2 Fig — Each point represents a metabolite. The horizontal coordinate represents the multiple change of the relative substances in the group (taking the logarithm of base 2). The vertical coordinate represents the P value of T test (taking the logarithm of base 10). The size of the scatter point represents the VIP value of the PLS-DA model. The larger the scatter point, the larger the VIP value. Significantly up-regulated metabolites are shown in red, significantly down-regulated metabolites are shown in blue, and non-significantly differentiated metabolites are shown in gray. (A) I6 vs. C6 group. (B) I15 vs. C15 group. (C) I30 vs. C30 group. (D) I60 vs. C60 group. (TIF) [file ppat.1011893.s004.tif]

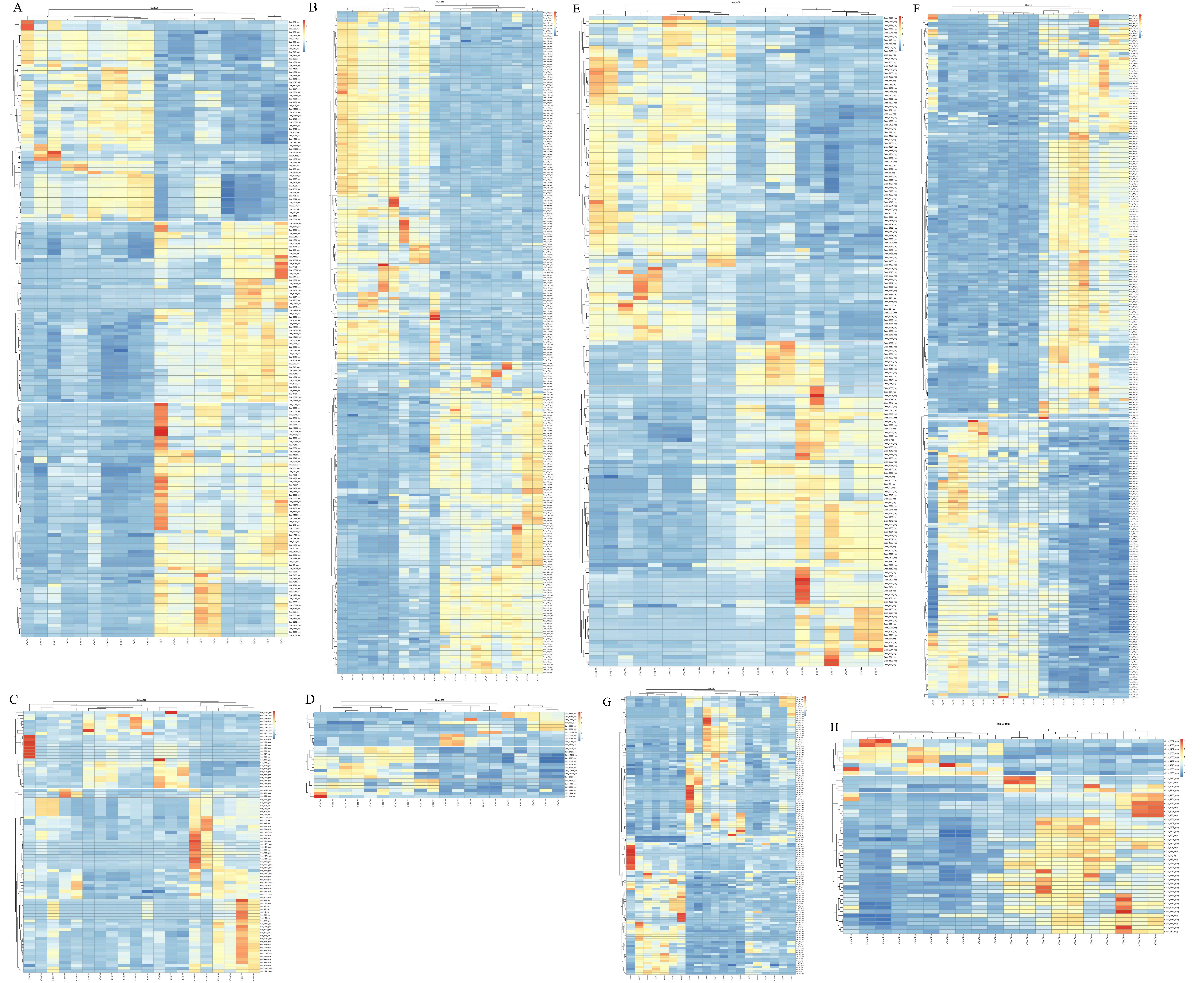

Supplement: S3 Fig — Each column represents a different sample and each row represents a different metabolite. Red indicates high expression metabolites, blue indicates low expression metabolites. Profiles of serum metabolites from positive (A-D) and negative ionic modes (E-H) were used to construct heat maps. Ten serum samples from infected mice and ten from control mice were analyzed at each time point. Red indicates that the metabolite had a higher expression level, and blue shows that the metabolite had a lower expression. (PDF) [file ppat.1011893.s005.pdf]
